# Investigating the Campylobacter jejuni Transcriptional Response to Host Intestinal Extracts Reveals the Involvement of a Widely Conserved Iron Uptake System

**DOI:** 10.1128/mBio.01347-18

**Published:** 2018-08-07

**Authors:** Martha M. Liu, Christine J. Boinett, Anson C. K. Chan, Julian Parkhill, Michael E. P. Murphy, Erin C. Gaynor

**Affiliations:** a Department of Microbiology and Immunology, University of British Columbia, Vancouver, BC, Canada; b Wellcome Sanger Institute, Wellcome Genome Campus, Hinxton, Cambridgeshire, United Kingdom; New York University

**Keywords:** Campylobacter jejuni, RNA-seq, acid resistance, chicken cecal extract, human fecal extract, iron transport, p19, streptomycin resistance, transcriptome

## Abstract

Campylobacter jejuni is a pathogenic bacterium that causes gastroenteritis in humans yet is a widespread commensal in wild and domestic animals, particularly poultry. Using RNA sequencing, we assessed C. jejuni transcriptional responses to medium supplemented with human fecal versus chicken cecal extracts and in extract-supplemented medium versus medium alone. C. jejuni exposed to extracts had altered expression of 40 genes related to iron uptake, metabolism, chemotaxis, energy production, and osmotic stress response. In human fecal versus chicken cecal extracts, C. jejuni displayed higher expression of genes involved in respiration (*fdhTU*) and in known or putative iron uptake systems (*cfbpA*, *ceuB*, *chuC*, and *CJJ81176_1649–1655* [here designated *1649–1655*]). The *1649–1655* genes and downstream overlapping gene *1656* were investigated further. Uncharacterized homologues of this system were identified in 33 diverse bacterial species representing 6 different phyla, 21 of which are associated with human disease. The *1649* and *1650* (*p19*) genes encode an iron transporter and a periplasmic iron binding protein, respectively; however, the role of the downstream *1651–1656* genes was unknown. A Δ*1651*–*1656* deletion strain had an iron-sensitive phenotype, consistent with a previously characterized Δ*p19* mutant, and showed reduced growth in acidic medium, increased sensitivity to streptomycin, and higher resistance to H_2_O_2_ stress. In iron-restricted medium, the *1651–1656* and *p19* genes were required for optimal growth when using human fecal extracts as an iron source. Collectively, this implicates a function for the *1649–1656* gene cluster in C. jejuni iron scavenging and stress survival in the human intestinal environment.

## IMPORTANCE

Direct comparative studies of C. jejuni infection of a zoonotic commensal host and a disease-susceptible host are crucial to understanding the causes of infection outcome in humans. These studies are hampered by the lack of a disease-susceptible animal model reliably displaying a similar pathology to human campylobacteriosis. In this work, we compared the phenotypic and transcriptional responses of C. jejuni to intestinal compositions of humans (disease-susceptible host) and chickens (zoonotic host) by using human fecal and chicken cecal extracts. The mammalian gut is a complex and dynamic system containing thousands of metabolites that contribute to host health and modulate pathogen activity. We identified C. jejuni genes more highly expressed during exposure to human fecal extracts in comparison to chicken cecal extracts and differentially expressed in extracts compared with medium alone, and targeted one specific iron uptake system for further molecular, genetic, and phenotypic study.

## INTRODUCTION

Campylobacter jejuni is a leading bacterial cause of human foodborne illness worldwide in both developed and developing nations (1, 2). This zoonotic bacterium asymptomatically colonizes the digestive tracts of wild and domestic animals, with population colonization rates measured as high as 86% (3–6). The most common reservoir of C. jejuni transmission to humans is poultry, particularly chickens. C. jejuni can colonize chicken ceca, which are two blind pouches at the junction of the small and large intestines, to concentrations as high as 2.5 × 10^9^ CFU/g cecal material without signs of pathology (7, 8). In humans, C. jejuni infection can result in sporadic cases or localized outbreaks of disease due to ingestion of contaminated meat, water, or unpasteurized milk and improper handling of animals (1, 9, 10). Accidental ingestion of as little as a few hundred bacterial cells can cause severe but typically self-limiting acute gastrointestinal illness ranging from mild to bloody diarrhea, nausea, and vomiting as early as 17 h postingestion and lasting from days to weeks (11–13). During human infection, C. jejuni colonizes the large intestine and can replicate to concentrations as high as 3.0 × 10^8^ CFU/g fecal material (12). C. jejuni infection can also cause long-term chronic inflammatory bowel diseases (e.g., Crohn’s disease, ulcerative colitis, and colorectal cancer) and autoimmune inflammatory demyelination diseases (e.g., Guillain-Barré and Miller-Fisher syndromes) in a small percentage of infected individuals (14, 15). Despite extensive study, it remains enigmatic why C. jejuni causes such acute human disease but harmlessly colonizes its zoonotic hosts, such as chickens.

The intestinal metabolome is a complex and dynamic system consisting of thousands of metabolites that both contribute and respond to intestinal health (16–18). The known metabolites, including short-chain fatty acids (SCFAs), organic acids, bile salts, lipids, amino acids, vitamins, and trace minerals (17), only account for a small fraction of the metabolome, with thousands of as yet unidentified metabolites that may play a role in intestinal health (19). Salmonella enterica serovar Typhimurium was recently shown to respond to human fecal metabolites by increasing the expression of genes related to metabolism, motility, and chemotaxis, reducing the expression of genes involved in host cell invasion, and reducing pathogen invasion of HeLa cells (19). The metabolomes of the chicken ceca and the human large intestine are expected to be dissimilar due to factors such as differences in diet, digestive system structure and function, host defense peptides, and the resident microbiome (20–22). These factors also contribute to the availability of trace metals and micronutrients, such as iron, that are essential for bacterial cell growth and host colonization (23–25). We thus hypothesized that C. jejuni exposed to human fecal extracts would respond by altering expression of genes specifically required for human infection.

In this study, we compared phenotypic and gene expression differences in C. jejuni cells exposed to medium containing sterile intestinal extracts of the disease-susceptible host (humans) versus the commensal host (chickens). Among the differentially expressed genes, we selected one putative and as yet uncharacterized iron uptake gene cluster (*CJJ81176_1649–1656*) for further study, as it was significantly more highly expressed during growth in medium with extracts compared to medium alone, and in particular in medium containing human fecal compared to chicken cecal extracts. This gene cluster is here referred to as “*1649–1656*,” and all genes discussed throughout the article will use the *CJJ81176* gene locus numbers and protein designations. Collectively, this work shows that C. jejuni responds differentially to metabolites present in the intestinal lumen of commensal versus susceptible hosts, provides a broad database repository of the C. jejuni transcriptional gene responses to these conditions, and shows that the 1649–1656 iron uptake system participates in numerous key phenotypes and thus may be integral to host infection, especially in humans.

## RESULTS

### C. jejuni cultured in medium containing chicken cecal or human fecal extracts compared to medium alone exhibited comparable levels of logarithmic growth, enhanced late-stage survival, and decreased biofilm formation.

**FIG 1:**
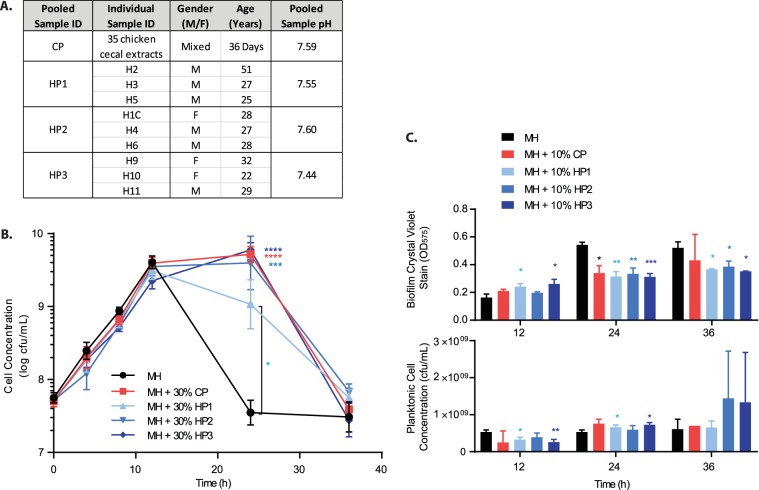
C. jejuni growth and biofilm formation in the presence of prepared extracts. (A) The four sterile pooled extracts (chicken pool [CP] and human pools 1, 2, and 3 [HP1, HP2, and HP3]) were prepared from 35 individual chicken cecal extracts and 9 human fecal extracts (H1C, H2, etc., human fecal extract numerical identifier; M/F, male/female). (B) C. jejuni growth in MH, MH plus 30% CP, and MH plus 30% HP1, HP2, and HP3 as measured by dilution plating. (C) C. jejuni biofilm formation over time with and without 10% extract as measured by the crystal violet assay (top) compared to the viable planktonic cell count (bottom). Error bars represent the standard deviation from 3 replicates. Statistical analysis for growth and biofilm formation was performed using the Student’s *t* test with Welch’s correction and compares MH plus extract conditions (CP, pink; HP1, light blue; HP2, medium blue; HP3, dark blue) to the MH control (black) for each time point. *, *P* < 0.05; **, *P* < 0.005; ***, *P* < 0.0005; ****, *P* < 0.0001.

Sterile pooled chicken cecal extract from 35 chickens (chicken pool [CP]) and three unique pools of human fecal extracts from 9 volunteers (human pools 1, 2, and 3 [HP1, HP2, and HP3]) were prepared by dilution of cecal/fecal material in sterile H_2_O and sterilization by filtration (Fig. 1A). Wild-type C. jejuni strain 81-176 was grown in Mueller-Hinton medium (MH) alone or MH supplemented with 30% CP, HP1, HP2, or HP3 to investigate the effects of extract exposure on *in vitro* growth and biofilm formation (Fig. 1B and C). The 30% supplement condition was selected for these analyses based on limited extract volume and C. jejuni sensitivity to high osmotic stress (26). C. jejuni exponential growth rates were comparable under each condition for the first 12 h after inoculation, with doubling times ranging between 1.9 and 2.2 h (Fig. 1B). At 24 h, C. jejuni cells grown in medium alone showed reduced viability, as expected under this growth condition, while C. jejuni cells incubated in medium containing either chicken cecal or human fecal extracts maintained significantly higher viability (Fig. 1B). Growth in extracts did not impact C. jejuni cell morphology (data not shown). Biofilm formation was assessed for C. jejuni exposed to medium alone, and to medium containing 10% chicken cecal or human fecal extracts (Fig. 1C). A concentration of 10% extract was selected to conserve extract, as preliminary testing showed no difference between levels of C. jejuni biofilm formation in medium containing 10% or 30% extracts (data not shown). After 12 h, biofilm formation in medium containing extracts was comparable to or slightly higher than that in medium alone. However, at 24 and 36 h, C. jejuni cells grown in medium containing extracts exhibited lower biofilm abundance than cells grown in medium alone. One exception was C. jejuni cells grown in medium containing 10% CP at 36 h, which did not show a significant difference. The reduced biofilm formation in medium containing extracts was not caused by poor growth, since planktonic cell concentrations were comparable or higher under these conditions, similar to observations in shaking broth noted above.

### C. jejuni exposed to host intestinal extracts responded by altering the expression of metabolism and iron uptake-related genes.

We used RNA sequencing to assess the transcriptional response of C. jejuni upon exposure to extracts. C. jejuni cells were incubated in medium alone, medium plus 30% CP, and medium plus 30% HP1, HP2, or HP3. RNA was collected after 20 min to assess transient differences in gene expression and after 5 h to assess adaptive homeostatic differences. Sample data from the 20 different conditions were grouped as outlined in Table S1 in the supplemental material, and two different evaluation conditions were analyzed in detail: (i) exposure to medium containing extract versus medium alone (referred to as extract versus medium—discussed in this section) and (ii) exposure to human fecal extracts versus chicken cecal extract (referred to as human versus chicken—discussed in the next section).

10.1128/mBio.01347-18.6 TABLE S1  Conditions for RNA sequencing, sequencing depth, accession numbers, and definition of sample grouping for fold change analysis. Download TABLE S1, DOCX file, 0.1 MB. Copyright © 2018 Liu et al. 2018 Liu et al. This content is distributed under the terms of the Creative Commons Attribution 4.0 International license.

C. jejuni cells exposed to medium containing either human or chicken extracts versus medium alone revealed 23 genes with >2-fold higher expression (Table 1) and 17 genes with >2-fold lower expression (Table 2). Of the genes showing higher expression after exposure to extracts, 13 were related to metabolism, nutrient uptake, and energy production (*aspA*, *dcuA*, *dcuB*, *0438*, *0439*, *0884*, *0885*, *1389–1391*, *metC*, *purB-2*, and *1570*) (27–29), and 3 encoded hypothetical proteins with no annotated function (*0204*, *0440*, and *1005*). Also demonstrating higher expression was a 7-gene cluster (*1649–1655*) that includes a homologue of an iron transporter in Escherichia coli (*1649*) (30), and *p19* (*1650*), encoding a periplasmic iron binding protein involved in iron transport (25, 31) (Table 1). Genes *0438* and *0439* showed the largest increase in expression both at 20 min (4.5- and 4.4-fold, respectively) and 5 h (11.8- and 12.1-fold, respectively). These genes (originally annotated by NCTC11168 locus tags *cj0414* and *cj0415*) are required for gluconate dehydrogenase activity, allowing C. jejuni to use gluconate as an electron donor (32). Of the 17 genes showing reduced expression after incubation in medium supplemented with extracts in comparison to medium alone, one was a cell surface adhesin (*peb3*), 9 were involved in metabolism, nutrient uptake, and energy production (*gltB*, *gltD*, *0580*, *0581*, *0685*, *0912*, *0941*, *0942*, and *1386*), 2 were involved in oxidative stress (*herA* and *1656*), and 4 encoded hypothetical proteins with no annotated function (*1006*, *1184*, *1185*, and *1657*) (Table 2). The gene exhibiting the greatest decrease during incubation in extracts was *peb3* at both 20 min (3.0-fold) and 5 h (6.2-fold). It encodes a cell surface glycoprotein adhesin that also imports phosphorylated compounds (33). PEB3 is a major antigenic protein during human infection, so rapid downregulation of this gene may be advantageous to host colonization (34, 35). A list of differentially expressed genes in C. jejuni cultured in medium with either human or chicken extracts in comparison to medium alone at each sampling time is in Table S2 in the supplemental material, and a Venn diagram of these genes is presented in Fig. S1 in the supplemental material.

10.1128/mBio.01347-18.3 FIG S1  Venn diagram of the number of genes differently expressed during growth in the presence of chicken extracts (CP) versus medium alone (M) and human extracts (HP) versus medium alone after 20 min and 5 h. See Text S2 for full legend. Download FIG S1, TIF file, 2.2 MB. Copyright © 2018 Liu et al. 2018 Liu et al. This content is distributed under the terms of the Creative Commons Attribution 4.0 International license.

10.1128/mBio.01347-18.7 TABLE S2  Changes in gene expression of C. jejuni cultured in the presence of chicken extracts versus medium alone and human extracts versus medium alone. Download TABLE S2, DOCX file, 0.1 MB. Copyright © 2018 Liu et al. 2018 Liu et al. This content is distributed under the terms of the Creative Commons Attribution 4.0 International license.

**TABLE 1:** C. jejuni genes showing higher expression (>2-fold) in medium containing extract than in medium alone

Locus tag	Gene name	Gene product function	20 min		5 h	
			Fold change	*P* value	Fold change	*P* value
Metabolism, nutrient uptake, and energy production						
CJJ81176_0122	*aspA*	Aspartate ammonia-lyase	3.2	6.13E−08	2.5	4.61E−05
CJJ81176_0123	*dcuA*	Anaerobic C_4_-dicarboxylate membrane transporter DcuA	2.9	2.79E−09	2.5	1.31E−06
CJJ81176_0438	*CJJ81176_0438*	Putative oxidoreductase subunit	4.5	1.97E−54	11.8	8.63E−149
CJJ81176_0439	*CJJ81176_0439*	Oxidoreductase, putative	4.4	4.54E−28	12.1	2.71E−80
CJJ81176_0697	*dcuB*	Anaerobic C_4_-dicarboxylate membrane transporter DcuB	2.1	3.52E−08	3.1	4.10E−19
CJJ81176_0884	*CJJ81176_0884*	Cytochrome *c* family protein, degenerate	2.1	2.47E−08	2.9	5.25E−19
CJJ81176_0885	*CJJ81176_0885*	Cytochrome *c*	2.4	5.34E−10	4.1	3.16E−27
CJJ81176_1389	*CJJ81176_1389*	DNA-binding protein	2.1	2.81E−05		
CJJ81176_1390	*CJJ81176_1390*	Reactive intermediate/imine deaminase	2.4	3.87E−02		
CJJ81176_1391	*CJJ81176_1391*	C_4_-dicarboxylate ABC transporter	2.5	1.31E−02		
CJJ81176_1392	*metC*	Cystathionine β-lyase	2.5	6.81E−03		
CJJ81176_1393	*purB-2*	Adenylosuccinate lyase	2.2	2.57E−02		
CJJ81176_1570	*CJJ81176_1570*	Anaerobic dimethyl sulfoxide reductase chain A			2.4	8.60E−03

Iron uptake						
CJJ81176_1649	*CJJ81176_1649*	Iron permease, FTR1 family			3.2	1.19E−04
CJJ81176_1650	*p19*	Periplasmic iron binding protein			3.8	1.01E−05
CJJ81176_1651	*CJJ81176_1651*	Membrane protein, putative			2.9	9.05E−04
CJJ81176_1652	*CJJ81176_1652*	ABC transporter, permease protein			3.5	6.02E−05
CJJ81176_1653	*CJJ81176_1653*	ABC transporter, permease protein			3.9	5.62E−06
CJJ81176_1654	*CJJ81176_1654*	ABC transporter, ATP-binding protein			4.3	1.14E−06
CJJ81176_1655	*CJJ81176_1655*	Thioredoxin, homologue			4.2	4.84E−06

Hypothetical proteins						
CJJ81176_0204	*CJJ81176_0204*	Hypothetical protein	2.9	1.85E−12	4.4	3.99E−26
CJJ81176_0440	*CJJ81176_0440*	Conserved hypothetical protein	2.4	1.69E−11	2.1	1.12E−08
CJJ81176_1005	*CJJ81176_1005*	Membrane protein, putative	2.1	1.11E−03		

**TABLE 2:** C. jejuni genes showing lower expression (>2-fold) in medium containing extract versus medium alone

Locus tag	Gene name	Gene product function	20 min		5 h	
			Fold change	*P* value	Fold change	*P* value
Adhesin						
CJJ81176_0315	*peb3*	Major antigenic peptide PEB3	−3.0	2.93E−06	−6.2	2.33E−17

Metabolism, nutrient uptake, and energy production						
CJJ81176_0033	*gltB*	Glutamate synthase, large subunit			−2.1	5.06E−04
CJJ81176_0035	*gltD*	Glutamate synthase, small subunit			−2.1	8.27E−31
CJJ81176_0580	*CJJ81176_0580*	C_4_-dicarboxylate ABC transporter			−3.4	5.77E−21
CJJ81176_0581	*CJJ81176_0581*	Amidohydrolase			−2.0	3.04E−07
CJJ81176_0685	*CJJ81176_0685*	Di/tripeptide transporter			−2.9	1.81E−26
CJJ81176_0912	*CJJ81176_0912*	Amino acid carrier protein			−2.5	1.88E−08
CJJ81176_0941	*CJJ81176_0941*	Sodium:alanine symporter	−2.5	7.05E−07		
CJJ81176_0942	*CJJ81176_0942*	Sodium:alanine symporter	−2.6	9.79E−07		
CJJ81176_1386	*CJJ81176_1386*	Fumarate reductase	−2.6	4.37E−02		

Chemotaxis						
CJJ81176_0109	*CJJ81176_0109*	Methyl-accepting chemotaxis protein			−2.4	1.13E−03

Oxidative response						
CJJ81176_0266	*herA*	Hemerythrin	−2.1	2.08E−02		
CJJ81176_1656	*CJJ81176_1656*	Thioredoxin family protein	−2.0	2.88E−02		

Hypothetical proteins						
CJJ81176_1006	*CJJ81176_1006*	Hypothetical protein			−2.3	1.43E−03
CJJ81176_1184	*CJJ81176_1184*	Hypothetical protein			−2.5	3.58E−04
CJJ81176_1185	*CJJ81176_1185*	Hypothetical protein			−2.6	9.42E−04
CJJ81176_1657	*CJJ81176_1657*	Hypothetical protein	−2.6	5.54E−31	−2.7	1.72E−32

### C. jejuni exposed to human fecal extracts versus chicken cecal extract had higher expression of iron uptake and formate dehydrogenase genes.

As noted above, we also compared C. jejuni gene expression during growth in medium supplemented with human fecal extracts to expression during growth in medium supplemented with chicken cecal extract. This identified 2 genes with >2-fold higher expression after 20 min and 12 genes with higher expression after 5 h (Table 3). No genes showing reduced expression were identified. The 2 genes with higher expression in human extracts after 20 min of exposure (*fdhT*, 2.4-fold; *fdhU*, 2.9-fold) increased further at 5 h (6.4- and 3.3-fold higher, respectively). FdhTU is involved in formate dehydrogenase activity and contributes to the invasion and intracellular survival of C. jejuni in intestinal epithelial cells (36, 37). The remaining 10 genes with 2.5- to 3.4-fold higher expression after 5 h are known or hypothesized to be involved in iron uptake and/or utilization (Table 3). The *cfbpA*, *ceuB*, and *chuC* genes encode parts of three different C. jejuni iron uptake systems: *cfbpA* encodes the periplasmic iron binding protein for the ferri-transferrin uptake system (38), *ceuB* encodes the periplasmic permease for the ferri-enterochelin uptake system (39), and *chuC* encodes part of the ABC transporter system for the heme uptake system (40, 41). None of the other components in these iron uptake systems showed higher expression in human fecal extracts versus chicken cecal extract (41). The remaining 7 genes were *1649–1655*, which were also more highly expressed in C. jejuni cells exposed to extracts in comparison to those exposed to medium alone and are described in more detail in the following sections.

**TABLE 3:** C. jejuni genes showing higher expression in medium containing human fecal extract in comparison to medium containing chicken cecal extract

Locus tag	Gene name	Gene product function	20 min		5 h	
			Fold change	*P* value	Fold change	*P* value
Respiration						
CJJ81176_1492	*fdhT*	Membrane protein, putative	2.9	8.27E−03	6.4	1.29E−10
CJJ81176_1493	*fdhU*	Conserved hypothetical protein	2.4	3.00E−02	3.3	8.96E−05

Iron uptake						
CJJ81176_0211	*cfbpA*	Iron ABC transporter, periplasmic iron-binding protein			2.8	1.56E−02
CJJ81176_1351	*ceuB*	Enterochelin ABC transporter, permease protein			2.9	1.58E−02
CJJ81176_1603	*chuC*	Hemin ABC transporter, ATP-binding protein, putative			3.4	2.63E−02
CJJ81176_1649	*CJJ81176_1649*	Iron permease, FTR1 family			2.7	3.44E−03
CJJ81176_1650	*p19*	Periplasmic iron binding protein			2.9	2.05E−03
CJJ81176_1651	*CJJ81176_1651*	Membrane protein, putative			2.6	7.86E−03
CJJ81176_1652	*CJJ81176_1652*	ABC transporter, permease protein			2.7	7.86E−03
CJJ81176_1653	*CJJ81176_1653*	ABC transporter, permease protein			2.7	7.86E−03
CJJ81176_1654	*CJJ81176_1654*	ABC transporter, ATP-binding protein			2.5	1.58E−02
CJJ81176_1655	*CJJ81176_1655*	Thioredoxin, homologue			2.5	3.59E−02

### Human fecal extracts contain higher total iron content than chicken cecal extract.

**FIG 2:**
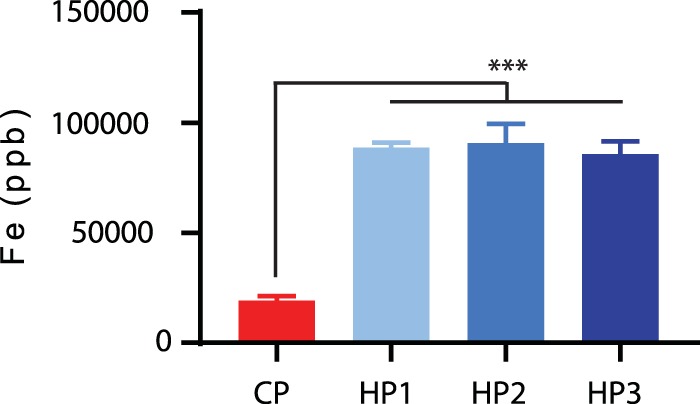
Iron concentration of pooled chicken and human extracts. The iron concentration of each pooled extract was measured using ICP-MS. Error bars represent the standard deviation from 2 duplicate tests. The significance was calculated by ANOVA. ***, *P* < 0.005.

Since the majority of the genes more highly expressed during growth in human fecal extracts were related to iron uptake, we hypothesized that cells grown in human fecal extracts were more iron starved than cells growing in chicken cecal extract. Quantification of the total iron present in extracts in all its forms was carried out using inductively coupled plasma mass spectrometry (ICP-MS). Unexpectedly, human extracts were found to contain approximately four times more iron than the chicken cecal extract (Fig. 2), suggesting the iron present is tightly chelated and requires specialized uptake systems.

### Homologues of *1649–1655* are widely conserved in the bacterial kingdom.

**FIG 3:**
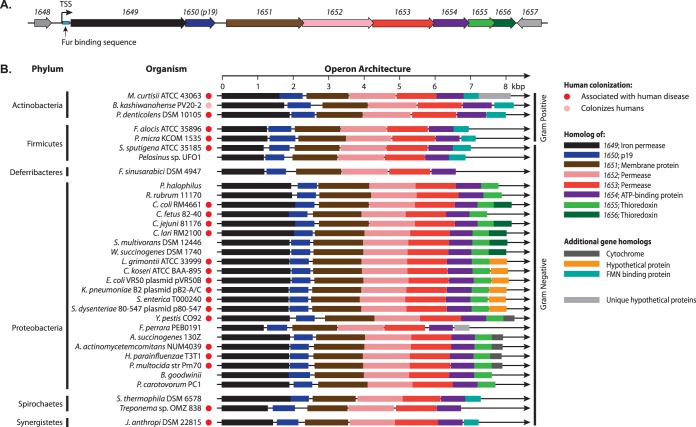
The *1649–1656* operon in C. jejuni and other bacterial species. (A) Proposed operon structure in C. jejuni wild-type strain 81-176, including flanking genes *1648* and *1657*. TSS, transcription start site. (B) Homologues found within representative bacterial species identified with blastp are shown to scale. The colored bars correspond to the genes described in the legend. Red and pink circles designate that the organism is associated with human disease or colonization, respectively. Organisms listed in alphabetical order: Actinobacillus succinogenes 130Z, Aggregatibacter actinomycetemcomitans NUM4039, Bifidobacterium kashiwanohense PV20-2, Brenneria goodwinii, Campylobacter coli RM4661, Campylobacter fetus 82-40, Campylobacter jejuni 81-176, Campylobacter lari RM2100, Citrobacter koseri ATCC BAA-895, Escherichia coli VR50(pVR50B), Filifactor alocis ATCC 35896, Flexistipes sinusarabici DSM 4947, Frischella perrara PEB0191, Haemophilus parainfluenzae T3T1, Jonquetella anthropi DSM 22815, Klebsiella pneumoniae B2(pB2-A/C), Leminorella grimontii ATCC 33999 (= DSM 5078), Mobiluncus curtisii ATCC 43063, Paracoccus halophilus, Parascardovia denticolens DSM 10105, Parvimonas micra KCOM 1535, Pasteurella multocida Pm70, Pectobacterium carotovorum PC1, *Pelosinus* sp. strain UFO1, Rhodospirillum rubrum ATCC 11170, Salmonella enterica serovar Typhimurium T000240, Selenomonas sputigena ATCC 35185, Shigella dysenteriae 80-547(p80-547), Spirochaeta thermophila DSM 6578, Sulfurospirillum multivorans DSM 12446, *Treponema* sp. strain OMZ 838, Wolinella succinogenes DSM 1740, and Yersinia pestis CO92.

The increased expression of the *1649–1655* genes suggested that they are important in the C. jejuni response to extracts, especially human fecal extracts. The Escherichia coli homologue (FetM) of 1649 is a ferrous iron permease (30), and the periplasmic protein 1650 (P19) has been characterized as an iron transporter in C. jejuni (25), E. coli (30), *Bordetella* (42), and Yersinia pestis (43). However, no prevalence or functional studies have been reported on the downstream genes (*1651–1656*) or homologues. As determined by conserved domain analyses and genome annotation, *1651–1655* were predicted to consecutively encode an inner membrane protein (1651), two inner membrane permeases (1652 and 1653), a cytoplasmic ATPase (1654), and a periplasmic thioredoxin (1655) (Fig. 3A). Also, one downstream gene (*1656*) overlaps with *1655* by 35 bp, encodes a second putative periplasmic thioredoxin (1656), and is assumed to be part of the same gene cluster (Fig. 3A). This set of 8 genes (*1649–1656*) is carried directly downstream of a Fur box and has one putative primary transcription start site located 54 bases upstream of the *1649* start codon, suggesting that these genes are transcribed as part of an operon (Fig. 3A) (44). The gene upstream of *1649* (*1648*, encoding a hypothetical protein) ends 350 bp before the start of *1649*, and the gene downstream of *1656* (*1657*, also encoding a hypothetical protein) is carried on the opposite strand. Each of these flanking genes has its own primary transcription start sites and is thus unlikely part of the proposed *1649–1656* operon (44).

Protein BLAST analyses showed that the 1649–1656 system is widely conserved in the bacterial kingdom. Components of the system are found in 22 of 28 fully sequenced *Campylobacter* species. Of the species lacking components of this system, C. avium, C. helveticus, C. hepaticus, and C. ornithocola have not been associated with human illness, and C. lanienae and C. sputorum are infrequently found by PCR in stools of both healthy and diarrhetic humans (45, 46). We also identified representative homologues of 1649–1656 in 33 phylogenetically diverse bacterial species with available complete genome sequences (Fig. 3B; see Table S3 in the supplemental material). Homologous gene products were most commonly found in members of the *Proteobacteria*, but were also present in *Actinobacteria*, *Firmicutes*, *Deferribacteres*, *Spirochaetes*, and *Synergistes*. Genes making up the operon were chromosomally carried in 30 of the 33 bacterial species and on plasmids in *Escherichia*, *Klebsiella*, and *Shigella*. Homologues of *1649–1656* were found in both Gram-positive and Gram-negative bacteria colonizing and infecting humans (22 out of 33 species identified), cows (Actinobacillus succinogenes and Wolinella succinogenes [47, 48]), honey bees (Frischella perrara [49]), and plants (Brenneria goodwinii and Pectobacterium carotovorum [50, 51]), as well as environmental isolates from water (Flexistipes sinusarabici, Paracoccus halophilus, and Rhodospirillum rubrum [52–54]), soil (*Pelosinus* sp. and Sulfurospirillum halorespirans [55, 56]), and thermal sites (Spirochaeta thermophila [57]). With respect to human colonizers, most bacterial species identified were associated with diseases such as gastroenteritis (e.g., Campylobacter jejuni), periodontal disease (e.g., Filifactor alocis [58]), urinary tract infection (e.g., Leminorella grimontii [59]), vaginosis (e.g., Mobiluncus curtisii [60]), and spondylodiscitis (e.g., Parvimonas micra [61]).

10.1128/mBio.01347-18.8 TABLE S3  Homologues of the C. jejuni 1649–1656 system. Download TABLE S3, DOCX file, 0.1 MB. Copyright © 2018 Liu et al. 2018 Liu et al. This content is distributed under the terms of the Creative Commons Attribution 4.0 International license.

The organization of the operon and predicted gene products in each of the 33 bacterial species were examined. All 33 species carried homologues of the first six genes (*1649–1654*) with the same gene order, similar gene lengths, and comparable spacing between genes (Fig. 3B). While most members of the *Proteobacteria* carried a predicted thioredoxin gene as the seventh gene in the cluster, members of the *Actinobacteria*, *Firmicutes*, *Spirochaetes*, and *Synergistetes* encoded a predicted flavin mononucleotide (FMN) binding protein. Although the thioredoxins may not be directly involved in iron uptake, both thioredoxins and FMN binding proteins can serve as a source of periplasmic or extracytoplasmic reduction potential to reduce ferric iron to the ferrous form for transport into the cell. In *Proteobacteria* without a thioredoxin encoded by the eighth gene, a gene coding for either a cytochrome or a hypothetical protein was present. The high genetic conservation of this system in multiple bacterial phyla suggests that, minimally, the first 7 gene products form one functional system, which is further investigated in the following sections.

### The *1651–1656* genes are involved in iron acquisition.

**FIG 4:**
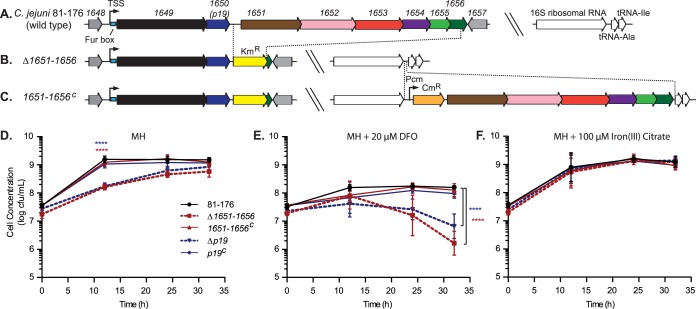
Construction of the Δ*1651–1656* mutant and complemented strains (*1651–1656*^*C*^) and their growth in iron-limiting and iron-supplemented media compared to the Δ*p19* and *p19*^C^ strains. (A) The wild-type *1649–1656* gene locus, including the upstream and downstream genes as well as the 16S rRNA region used for complementation. TSS, transcription start site; blue box, Fur binding sequence (Fur box). (B) The Δ*1651–1656* mutant was constructed by replacing *1651–1656* with a kanamycin resistance cassette (Km^r^). The region deleted is indicated. (C) Using the integrative vector pRRC (85), the *1651–1656* gene cluster was inserted into a noncoding region between the 16S rRNA and tRNA-Ala to generate the *1651–1656*^*C*^ complement. The *1651–1656* genes in the complemented strain are under the control of a chloramphenicol promoter (Pcm) upstream of the chloramphenicol resistance marker (Cm^r^) used for selection. Growth over time of the C. jejuni wild-type (black solid line), Δ*1651–1656* (red dotted line), *1651–1656*^C^ (red solid line), Δ*p19* (blue dotted line), and *p19*^C^ (blue solid line) strains in MH (D), iron-depleted MH containing 20 µM DFO (E), and iron-supplemented MH containing 100 µM iron(III) citrate (F) was measured by dilution plating. Error bars represent the standard deviations from 3 replicates from a total of 3 experiments. Statistical comparison of the Δ*1651–1656* and Δ*p19* mutants versus the wild-type control was performed using the Student’s *t* test with Welch’s correction, where indicated. ****, *P* < 0.0001.

To determine if the *1651–1656* genes were involved in iron acquisition similar to *1649* and *p19*, deletion (Δ*1651–1656*) and complemented (*1651–1656*^*C*^) C. jejuni strains were constructed (Fig. 4). The *p19* (*1650*) deletion mutant (Δ*p19*) and complemented (*p19*^*C*^) strains were used as controls since P19 is well conserved and is known to be involved in iron transport (25). The Δ*p19* and Δ*1651–1656* mutants each exhibited lower growth rates than the wild-type 81-176 strain or the corresponding complements during growth in MH medium, but reached comparable final viable cell concentrations at 32 h (Fig. 4D). Iron depletion by addition of 20 µM desferroxamine (DFO), an iron chelator, resulted in a similar initial growth rate for the Δ*1651–1656* mutant, a modestly reduced initial growth rate for the Δ*p19* mutant, and significantly reduced viability for both mutants after 12 h compared to the wild-type and complemented strains (Fig. 4E). Growth rates in MH for each mutant could be restored to wild-type and complement levels by supplementation with 100 µM iron(III) citrate (Fig. 4F).

### The 1649–1656 iron uptake system is involved in the C. jejuni response to acid, streptomycin, and H_2_O_2_-mediated oxidative stress.

**FIG 5:**
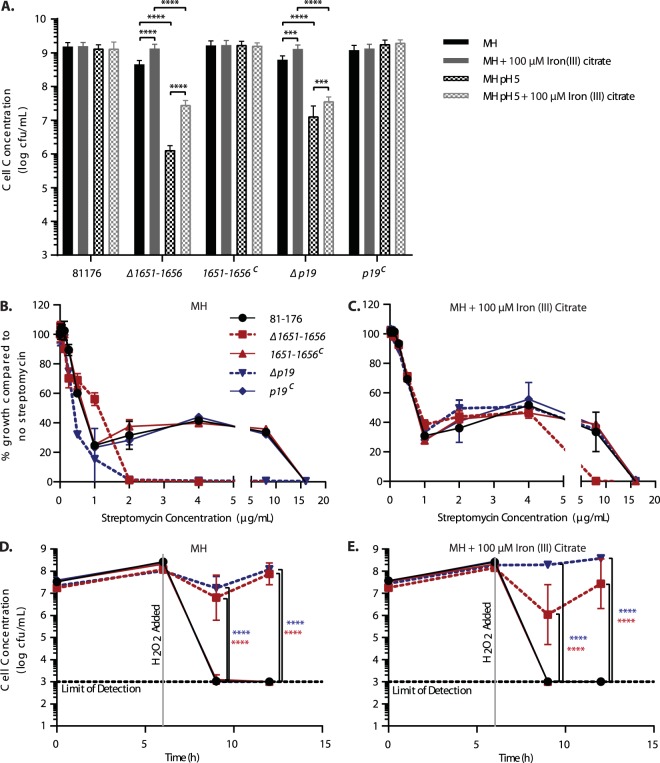
The role of 1651–1656 in C. jejuni survival under acid, oxidative (H_2_O_2_), and antibiotic (streptomycin) stress in comparison to P19. (A) Acid stress: comparison of growth in MH or in MH at pH 5 with and without 100 µM iron(III) citrate supplementation after 24 h (B and C) of antibiotic stress. Shown is the percentage of cell growth (based on OD_600_) in doubling dilutions of streptomycin compared to the no-streptomycin control when incubated in MH (B) and MH supplemented with 100 µM iron(III) citrate (C) for 48 h. (D and E) Oxidative stress. Shown is the viability of C. jejuni strains over 6 h after H_2_O_2_ addition in MH alone (D) or MH supplemented with iron(III) citrate (E). C. jejuni strains are shown as the wild type (black solid line), the Δ*1651–1656* mutant (red dotted line), the *1651–1656*^*C*^ complement (red solid line), the Δ*p19* mutant (blue dotted line), and the *p19*^C^ complement (blue solid line). Error bars represent the standard deviation from 3 replicates from a total of 3 experiments. Statistical comparison of the Δ*1651–1656* and Δ*p19* mutants to the wild-type 81-176 strain was performed using Student’s *t* test with Welch’s correction. ***, *P* < 0.0005; ****, *P* < 0.0001.

C. jejuni wild-type strain 81-176, the Δ*p19* and Δ*1651–1656* mutant strains, and the respective complements were exposed to acid (pH 5), antibiotic, and oxidative stress (1 mM H_2_O_2_) conditions to determine whether this iron uptake system is important for C. jejuni stress survival (Fig. 5). To test acid tolerance, cells were inoculated into MH at neutral pH or MH at pH 5 with and without supplementation with 100 µM iron(III) citrate and grown for 24 h. The Δ*p19* and Δ*1651–1656* mutants exhibited a 2- to 3-log reduction in growth at pH 5 in comparison to neutral pH, while growth of the wild-type and complement strains was unaffected by the pH 5 condition (Fig. 5A). The acid sensitivity phenotype of the mutants was observed in media both with and without 100 µM iron(III) citrate supplementation; however, iron supplementation partially restored growth of the mutants (Fig. 5A). Iron is more soluble at lower pH, which may contribute to the improved growth of mutants in acidic medium supplemented with iron(III) citrate.

To assess whether this iron uptake system was required for antibiotic resistance, the MICs of different antibiotics representing multiple classes (aminoglycosides, amphenicols, β-lactams, cationic peptides, fluoroquinolones, and macrolides) were screened for wild-type and Δ*1651–1656* deletion mutant strains using standard doubling dilution assays. Most classes showed ≤2-fold differences from wild type, except aminoglycosides, the most notable of which was streptomycin (STM) (data not shown). To test STM susceptibility in more depth, cells were inoculated into MH or MH supplemented with 100 µM iron(III) citrate containing no STM or doubling dilutions of STM from 0.02 to 16 µg/ml and grown for 48 h. In iron-unsupplemented MH, the MICs observed for the Δ*1651–1656* (2 µg/ml) and Δ*p19* (2 µg/ml) mutants were 8 times lower than the STM MIC for the wild-type and complemented strains (16 µg/ml). Supplementation of MH with excess iron restored the MICs of the Δ*1651–1656* and Δ*p19* mutants (8 and 16 µg/ml, respectively) to wild-type levels (16 µg/ml). To expand our analysis of STM sensitivity, cell densities (optical density at 600 nm [OD_600_]) were measured under increasing concentrations of STM both with and without iron supplementation (Fig. 5B and C). A bimodal growth phenotype was observed for wild-type and complemented strains both with and without iron supplementation (Fig. 5B and C). This bimodal phenotype consisted of STM-sensitive growth between 0 and 1 µg/ml, where cell density decreased rapidly from 100% to <40% compared to growth with no STM, and then STM-tolerated growth between 1 and 8 µg/ml. Unlike the wild-type and complemented strains, the Δ*1651–1656* and Δ*p19* mutants exhibited monomodal growth under increasing STM concentrations in iron-limited MH, with both strains becoming undetectable at 2 µg/ml STM (Fig. 5B). Iron supplementation restored bimodal growth for both mutants (Fig. 5C).

To examine if this operon had an effect on oxidative stress, wild-type strain 81-176, the Δ*p19* and Δ*1651–1656* mutants, and their respective complements were grown in medium alone or medium supplemented with 100 µM iron(III) citrate for 6 h, at which time H_2_O_2_ was added to a final concentration of 1 mM. Cell viability was monitored at 3 h and 6 h after H_2_O_2_ addition. The mutants were more resistant to H_2_O_2_ stress than the wild-type or complemented strains when grown in MH and in iron-supplemented MH, remaining viable at 3 h and 6 h post-H_2_O_2_ addition, whereas the wild-type and complemented strains were no longer recoverable by 3 h (Fig. 5D and E).

### The 1649–1656 system is required for optimal iron acquisition from human and chicken extracts.

**FIG 6:**
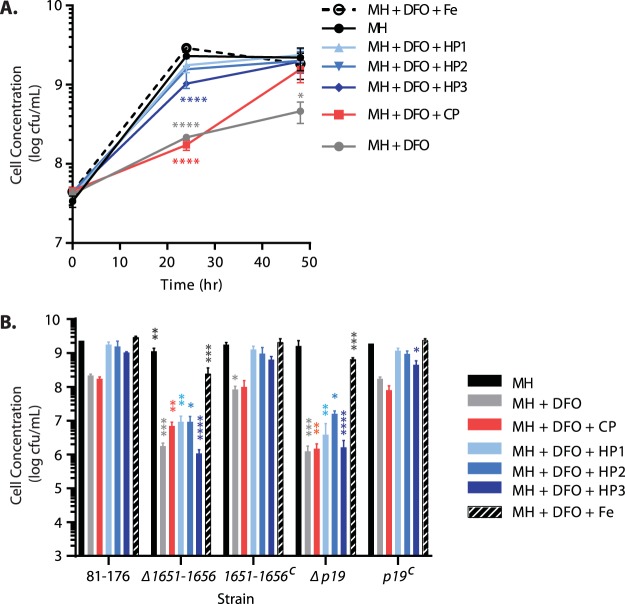
Growth of C. jejuni strains in iron-depleted medium and with extract supplementation. Shown is C. jejuni growth in control medium (MH; black), iron-limited medium (MH plus 15 µM DFO [gray]), and iron-limited medium supplemented with extracts (10% CP/HP1/HP2/HP3 [CP, pink; HP1, light blue; HP2, medium blue; HP3, dark blue) or iron(III) citrate (10 µM [black dashed/striped). (A) Growth of wild-type C. jejuni over 48 h. Statistical significance was calculated in comparison to the MH control. (B) Growth of wild-type, deletion mutant, and complemented strains at 24 h. Statistics were calculated in comparison to wild-type strain 81-176 for each growth condition. For both panels A and B, *P* values are as follows: *, *P* < 0.05; **, *P* < 0.005; ***, *P* < 0.0005; ****, *P* < 0.0001.

To determine whether the 1649–1656 system is involved in acquisition of iron found in extracts, we grew the wild-type and mutant strains in iron-limited MH (15 µM DFO) or iron-limited MH supplemented with 10% chicken or human extracts. Growth in MH and iron-limited MH supplemented with 10 µM iron(III) citrate was included as a control. The DFO concentration of 15 µM was selected to allow optimal C. jejuni responsiveness to iron supplementation based on optimization testing, and the iron(III) citrate concentration of 10 µM was selected to approximate the maximum amount of iron expected in the extract samples. After 24 h, wild-type and complemented C. jejuni strains showed reduced growth under iron restriction in comparison to the MH control, which was rescued by supplementation with human extracts or iron(III) citrate (Fig. 6A; see Fig. S2A to F in the supplemental material). Notably, supplementation with chicken cecal extract was not sufficient to restore wild-type growth at 24 h, but did improve wild-type growth at 48 h in comparison to iron-limited medium alone (Fig. 6A; Fig. S2C and F). These results suggest that wild-type C. jejuni can utilize iron present in the extracts and that more accessible iron is present in human fecal than in chicken cecal extracts. The Δ*p19* and Δ*1651–1656* deletion strains were also deficient for growth in iron-restricted MH. However, supplementation with human fecal extracts did not restore bacterial growth to levels observed in MH alone or iron-limited MH supplemented with iron(III) citrate (Fig. 6B; Fig. S2A to F). This demonstrated that the 1649–1656 system is required for optimal acquisition of iron from human fecal extracts.

10.1128/mBio.01347-18.4 FIG S2  Growth of C. jejuni strains in iron-depleted medium and with extract supplementation over 48 h. See Text S2 for full legend. Download FIG S2, EPS file, 1.2 MB. Copyright © 2018 Liu et al. 2018 Liu et al. This content is distributed under the terms of the Creative Commons Attribution 4.0 International license.

## DISCUSSION

The ability of C. jejuni to colonize both chicken ceca and human large intestines to high concentrations (7, 8, 12) is reflected in this study, where supplementation of MH with extracts prolonged C. jejuni survival in broth culture. The lower biofilm formation in extracts, which may provide additional nutrients, is consistent with studies indicating that C. jejuni biofilm formation is less abundant in richer media (62). We collected intestinal extracts using water as a diluent, which preferentially extracts polar compounds such as amino acids, carbohydrates, alcohols, and short-chain fatty acids (63). Accordingly, C. jejuni cells exposed to these extracts responded by modulating expression of many genes related to metabolism, nutrient uptake, energy production, and chemotaxis. Other diluents used to prepare fecal extracts, such as methanol, acetonitrile, and ethyl acetate, preferentially extract aromatic compounds and lipids (16, 19, 63, 64). Future assessments may benefit from using different diluents to determine whether additional biologically active metabolites also impact pathogen response.

In this study, C. jejuni exposed to extracts, and in particular human fecal extracts, showed higher expression of genes related to iron uptake and utilization than C. jejuni in medium alone. A recent study of C. jejuni gene expression in human feces postcolonization also found that genes involved in iron acquisition were more highly expressed: notably, 6 out of 8 genes in the *1649–1656* operon were identified (65). Iron is an essential micronutrient for most organisms, with many producing high-affinity iron chelating proteins and siderophores to scavenge iron. C. jejuni does not produce siderophores, but does possess at least 5 distinct systems to import iron from exogenous chelators (41). These systems transport iron bound to enterobactin (CfrA, CfrB, CeuBCDE [66, 67]), heme (ChuABCDZ [40]), lactoferrin/transferrin (CtuA, CfbpABC, and ChaN [68]), ferrous ions (FeoB [69]), and potentially bound to rhodotorulic acid (Cj1658−1663 in strain 11168, which are homologues of 1649–1654 in strain 81-176 [31]). C. jejuni globally regulates expression of the majority of its iron uptake systems through the iron-responsive Fur regulator (67, 70, 71). The specific increase in *1649–1655* expression during exposure to human extracts and during human colonization, rather than a global response to low iron availability, suggests that other regulatory mechanisms target this system in addition to the Fur-based response. Furthermore, *1656*, which shares an overlapping reading frame with *1655*, actually showed a small decrease in expression during C. jejuni exposure to extracts, which may suggest even more complex regulation mechanisms at play.

It remains to be determined why the *1649–1655* operon was more highly expressed during C. jejuni exposure to extracts, especially human extracts, which contain more iron than the chicken cecal extracts. One hypothesis is that the iron present in the extracts, particularly human fecal extracts, is chelated to a compound that is specifically recognized by this iron uptake system. Indeed, in medium depleted of iron, supplementation with human extracts was able to fully restore growth of wild-type C. jejuni but not of the Δ*p19* or Δ*1651–1656* mutant. The 1649–1656 system was suggested to import iron bound to rhodotorulic acid, a fungal hydroxamate siderophore (reported as unpublished data in reference 31). However, other studies show that C. jejuni is unable to utilize iron bound to rhodotorulic acid for growth (72, 73); therefore, other chelators must be considered.

In Yersinia pestis, mutations in *fetMP* (homologues of C. jejuni *1649* and *p19*) resulted in iron-deficient growth that could only be restored when the downstream genes *y2367* to *y2362* (homologues of C. jejuni 81-176 genes *1651–1655*) were included in the complementation strain (43). Here, genes downstream of *p19* (*1651–1656*) were also required for C. jejuni growth under iron-restricted conditions. Furthermore, the widespread distribution and genetic conservation of the *1649–1656* cluster in bacteria with a variety of host preferences (e.g., humans, plants, cows, and bees) and ecologic niches (e.g., freshwater, seawater, soil, and thermal site) suggests that this entire system is used for iron uptake in many contexts. An analysis of the diversity and phylogenetic evolution of this iron uptake system, together with follow-up genetic and biochemical analyses, will allow better understanding of its role in bacteria.

Iron uptake in C. jejuni may be generally related to acid tolerance and antibiotic survival. Transcriptomic and proteomic studies have shown that this iron uptake system, in addition to several other iron uptake genes (*cfbpA*/*B*, *ceuE*, and *chuZ*), was more highly expressed upon C. jejuni exposure to acid stress (74, 75). In addition, deletion of *fur* increased C. jejuni sensitivity to acidic conditions (76). Paradoxically, however, C. jejuni acid sensitivity caused by deletion of *p19*, *1651–1656*, and *fur* was independent of iron availability in the growth medium, further indicating that additional regulatory mechanisms must be considered. Variability in iron availability and disturbance of bacterial iron homeostasis have also been shown to impact antibiotic efficacy in various bacteria (77). However, since the C. jejuni 81-176 strain has not been reported to carry streptomycin resistance genes, the mechanism of streptomycin tolerance and potential contribution of iron to this extended tolerance are unknown. The interplay between acid stress and iron homeostasis remains an interesting avenue for future research. Additional information pertaining to how C. jejuni responds to these stresses and the implications for resistance to streptomycin can be gained from genomewide expression analyses of C. jejuni under a combination of acid and iron stresses.

Unexpectedly, the 1649–1656 system adversely affected the C. jejuni response to H_2_O_2_ independent of the addition of iron. C. jejuni responds to oxidative stress and iron limitation by inducing expression of a common subset of genes (e.g., *katA*, *sodB*, and *ahpC*) that are controlled by both the Fur and PerR regulons (78, 79). Thus, loss of the 1649–1656 iron uptake system may cause constitutive cellular stress that elevated the baseline expression of genes contributing to oxidative stress tolerance. Alternatively, the presence of iron and copper binding proteins in the 1649–1656 system may directly render C. jejuni more sensitive to H_2_O_2_-mediated cell death by formation of highly reactive and damaging intermediates upon exposure to H_2_O_2_ (25, 80). Exploring whether related conditions such as nitrosative stress yield similar results would be interesting, as would examining the effects of combining the Δ*1651–1656* or Δ*p19* mutations with mutations in genes known to be required for oxidative stress survival.

**FIG 7:**
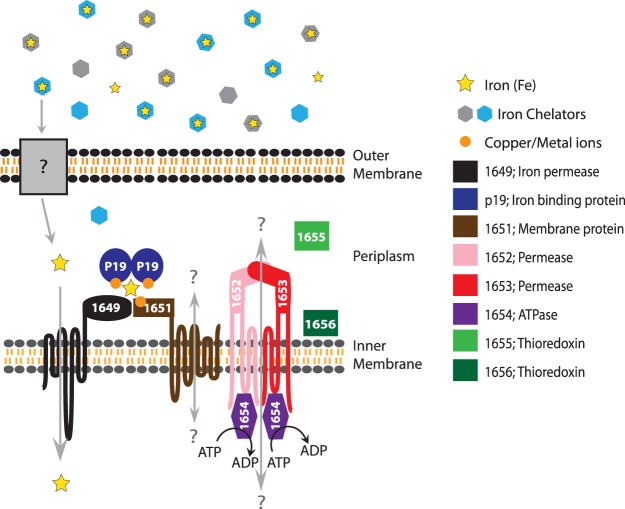
Model of the C. jejuni 1649–1656 iron uptake system. We hypothesize that this iron acquisition system imports iron (yellow stars) that is bound to a specific, but as yet unidentified, chelator (light blue hexagons). Gray hexagons represent other iron chelators. Since the gene cluster does not appear to encode an outer membrane receptor, Chan et al. suggested that iron is transported across the outer membrane by an as yet unidentified receptor (gray box) before binding to P19 (dark blue) in the periplasm (25). During iron uptake, iron-bound P19 may interact with the periplasmic metal binding domains of 1651 (brown), followed by an iron reduction step involving the 1655 and 1656 thioredoxins (light and dark green), prior to being transported into the cytoplasm via the 1649 inner membrane transporter (black). The substrates transported by the 1651 membrane protein and the 1652 and 1653 permeases (pink and red), with the help of the 1654 ATPase (purple), are unknown. We hypothesize that the chelated form of iron acquired by this system is more abundant in human than in chicken intestinal environments.

An updated model of the 1649–1656 iron uptake system is proposed based on bioinformatic analyses, showing the presence of signal peptides, transmembrane regions, and protein domains (Fig. 7). The *1649–1656* gene products are hypothesized to form one functional system based on comparable phenotypes observed between the Δ*1651–1656* and Δ*p19* mutants and conservation of the gene cluster in multiple bacterial phyla. Of note, the 1651 membrane protein has a periplasmic DUF2318 domain, encoding a conserved CxxC-x(13)-CxxC-x(14,15)-C motif (see Fig. S3A and B in the supplemental material). Conserved cysteines with regular spacing are often observed in iron-sulfur cluster binding domains, such as those seen in the Fer2 and Fer4 families (Fig. S3C and D). Furthermore, we found that genes encoding DUF2318-containing proteins almost always mapped to gene clusters with homologues of *1649–1656* in different bacterial species, indicating association with this bacterial iron uptake system. Ongoing experiments aimed toward single gene deletions and domain or residue specific substitutions should help determine the identity of transported substrates, protein-protein interactions, and mechanism(s) of iron transport.

10.1128/mBio.01347-18.5 FIG S3  The CxxC-x(13)-CxxC-x(14,15)-C motif of the DUF2318 domain in 1651. See Text S2 for full legend. Download FIG S3, TIF file, 2.5 MB. Copyright © 2018 Liu et al. 2018 Liu et al. This content is distributed under the terms of the Creative Commons Attribution 4.0 International license.

In sum, this study is the first to compare the bacterial transcriptional and functional responses to gut extracts from a disease-susceptible host (humans) and a zoonotic host (chicken). RNA sequencing identified genes that may be important for C. jejuni to colonize both chickens and humans, with subsequent biological analyses demonstrating that the 1649–1656 iron uptake system was necessary for C. jejuni growth under iron-depleted conditions, impacted growth and survival under specific stress-related conditions, and was required for acquisition of iron from human fecal extracts. Given the system’s conservation in a wide range of bacteria, the majority of which colonize and infect humans, this work should be broadly relevant to the general bacteriology and pathogenesis communities as well. Collectively, we showed that investigation of pathogen gene expression in response to exposure to the host metabolome, together with follow-up genotypic and phenotypic analyses, is not only an informative method of studying host-pathogen interactions but should improve our understanding of the mechanisms employed by pathogens to cause disease.

## MATERIALS AND METHODS

### Ethics statement.

Written and informed consent was obtained from all human fecal sample donors as described in ethics application H14-00859, which was approved by the University of British Columbia Clinical Research Ethics Board in Vancouver, Canada.

### Bacterial strains and growth conditions.

C. jejuni strain 81-176, a pathogenic and common laboratory reference strain, was used in this study (9). The C. jejuni Δ*1651–1656* mutant strain and corresponding *1651–1656*^*C*^ complemented strain were constructed as described in Text S1 in the supplemental material, using primers listed in Table S4 in the supplemental material. The Δ*p19* deletion and complemented (*p19*^*C*^) strains were obtained from reference 25. C. jejuni strains were grown in MH (Oxoid) broth or agar (1.5% [wt/vol]). MH was supplemented with antibiotics where appropriate: vancomycin (V; 10 µg/ml), trimethoprim (T; 5 µg/ml), kanamycin (Km; 50 µg/ml), and chloramphenicol (Cm; 20 µg/ml). Agar plates and standing cultures were incubated at 38°C under microaerobic and capnophilic conditions (12% CO_2_ and 6% O_2_ in N_2_) in a Sanyo tri-gas incubator. Shaking broth cultures (including 15- to 18-h overnight cultures) were incubated microaerobically in airtight containers with the Oxoid CampyGen atmosphere generation system at 38°C and shaken at 200 rpm. Cell concentration was measured by dilution plating; the limit of detection for this method is 10^3^ CFU/ml. Escherichia coli strain DH5α (Invitrogen) was used for cloning. E. coli was grown aerobically at 37°C and shaken at 200 rpm in Luria-Bertani broth (LB; Sigma) or incubated aerobically at 37°C on LB agar (1.5% [wt/vol]) supplemented with Cm (20 µg/ml) and Km (50 µg/ml) for selection, as required.

10.1128/mBio.01347-18.1 TEXT S1  Supplemental methods. Download TEXT S1, DOCX file, 0.1 MB. Copyright © 2018 Liu et al. 2018 Liu et al. This content is distributed under the terms of the Creative Commons Attribution 4.0 International license.

10.1128/mBio.01347-18.9 TABLE S4  Primers used in this study. Download TABLE S4, DOCX file, 0.1 MB. Copyright © 2018 Liu et al. 2018 Liu et al. This content is distributed under the terms of the Creative Commons Attribution 4.0 International license.

### Growth and biofilm formation in extracts.

Human fecal and chicken cecal extracts were prepared as described in Text S1. For liquid growth cultures, overnight log-phase C. jejuni cells were inoculated at an OD_600_ of 0.005 in a final volume of 1 ml in MH supplemented with trimethoprim and vancomycin (MH-TV) or MH-TV plus 30% extract. MH-TV plus 30% extract conditions were prepared by combining 0.5 ml of 2× MH-TV containing C. jejuni at an OD_600_ of 0.01, 0.3 ml of extract (CP, HP1, HP2, or HP3), and 0.2 ml of sterile ultrapure H_2_O (0.5 ml for the control MH-TV condition). Cultures were incubated microaerobically at 38°C and shaken. The cell concentration was measured by dilution plating at the indicated times. For biofilm formation, overnight log-phase C. jejuni cells were inoculated at an OD_600_ of 0.02 into 1 ml of MH-TV or MH-TV containing 10% extract (CP, HP1, HP2, HP3, or H_2_O for the control) in borosilicate glass tubes. The cultures were incubated microaerobically at 38°C without agitation. The planktonic cell concentration was measured by dilution plating 20 µl of culture sampled from 2 mm below the liquid surface. The biofilms were quantified as detailed in Text S1. Experiments were performed in triplicate.

### RNA preparation.

C. jejuni overnight cultures were reinoculated into fresh MH-TV broth at an OD_600_ of 0.04 and incubated microaerobically shaking at 38°C for 4 to 5 h in order to obtain optimal log growth (approximate OD_600_ of 0.2). C. jejuni cells were inoculated into MH-TV or MH-TV plus 30% extract (CP, HP1, HP2, or HP3) to a final volume of 1 ml at an OD_600_ of 0.25 for the 20-min conditions and OD_600_ 0.06 for the 5-h conditions in order to ensure comparable numbers of cells during harvest and RNA preparation. RNA was extracted from these 10 independent conditions performed in duplicate on separate days as outlined in Text S1. RNA from all samples was extracted by adding 1:10 (vol/vol) stop solution (5% phenol in ethanol) and was extracted as described previously (81). Samples were shipped to the Wellcome Trust Sanger Institute (WTSI), and RNA was sequenced and analyzed as described in Text S1.

### Total iron content of extracts.

ICP-MS was used to measure the iron content in human fecal and chicken cecal extracts. Briefly, 0.5 ml each of CP, HP1, HP2, and HP3 was added in duplicate into microcentrifuge tubes and dried overnight using a SpeedVac concentrator (DNA 120; Thermo) at low power. Samples were then analyzed using ICP-MS. Briefly, samples were digested in 1% nitric acid and heated in a closed Savillex vessel using a hot plate. Scandium (Sc) and indium (In) at 100 ppb were added as internal standards. ICP-MS was conducted using the PerkinElmer NexIon 300D ICP-MS instrument.

### Protein domains and homologues.

The Simple Modular Architecture Research Tool (SMART) in Genomic mode was used to determine protein domains, signal peptides, and PFAM domains for the amino acid sequences of 1649–1656 (http://smart.embl.de/) (82). Protter was used to visualize the protein placement in two-dimensional (2D) space (83). Homologues of 1649–1656 were identified using the NCBI online database with the blastn and blastp suites. To identify homologues in non-*Campylobacter* organisms, the “*Campylobacter* (taxid: 194)” group was excluded, and fully sequenced and annotated genomes of bacteria from each unique genus listed were downloaded from GenBank and visualized using Artemis. The amino acid sequences for each protein were aligned using MUSCLE and assessed for homology using MEGA 7.0.21 and BioEdit Sequence Alignment Editor, and conserved residues were visualized using the online tool Skylign (http://skylign.org/).

### Measuring growth under iron depletion, acid, streptomycin, and H_2_O_2_ stresses.

Log-phase C. jejuni cells of the 81-176, Δ*1651–1656*, *1651–1656*^*C*^, Δ*p19*, and *p19*^*C*^ strains from 15- to 18-h overnight cultures were inoculated at an OD_600_ of 0.005 into 3 ml of medium unless otherwise indicated. For growth under iron-depleted versus replete conditions, cells were inoculated into MH (unsupplemented), MH containing 20 µM ferric iron chelator DFO (iron poor), or MH supplemented with 100 µM iron(III) citrate (iron rich). For H_2_O_2_ stress, cells were inoculated into MH or MH plus 100 µM iron(III) citrate and incubated for 6 h, and then H_2_O_2_ (Sigma) was added to a final concentration of 1 mM H_2_O_2_. For acid stress, cells were inoculated into MH or MH at pH 5, prepared by adding 1 N HCl into MH and verified using a pH meter (SB20; VWR), with and without supplementation with 100 µM iron(III) citrate. For these growth experiments, cells were incubated shaking microaerobically at 38°C, and cell viability was measured by dilution plating at the indicated times. For antibiotic testing, cells were inoculated into 96-well plates at a final OD_600_ of 0.02 in 0.2 ml of MH or MH plus 100 µM iron(III) citrate containing doubling dilutions of streptomycin from 0.03 to 16 µM. The plates were incubated at 38°C for 48 h. The wells were mixed by pipetting and measured using the Varioscan Flash spectrophotometer at OD_600_. All experiments were performed in triplicate, with each experiment including three technical replicates.

### Measuring growth in iron-depleted medium supplemented with extracts.

Log-phase C. jejuni cells of the 81-176, Δ*1651–1656*, *1651–1656*^*C*^, Δ*p19*, and *p19*^*C*^ strains were inoculated at a final OD_600_ of 0.005 into 200 µl of MH, MH plus 15 µM DFO, MH plus 15 µMDFO plus 10% CP/HP1/HP2/HP3, or MH plus 15 µM DFO plus 10 µM iron(III) citrate in 96-well plates. Plates were incubated at 38°C microaerobically. At each time point, the wells were mixed, cell density was measured at OD_600_, and CFU measured by dilution plating 10 µl of each culture. Experiments were performed in triplicate.

### Statistics.

The *P* values for gene expression fold change derived from RNA sequencing data were calculated by the DESeq2 package. Briefly, DESeq2 uses the Benjamini-Hochberg (BH) adjustment to calculate the *p*_adj_ value, which represents the false-discovery rate of genes should the null hypothesis (i.e., no genes are affected) be true (84). Genes with *p*_adj_ values (shown as *P* values in Tables 1 to 3; see Table S2) lower than 0.05 were selected for this study. C. jejuni growth and biofilm data were analyzed and graphed using Graphpad Prism 7, and statistical differences were calculated using the Student’s *t* test. Statistical analysis of the ICP-MS data was calculated using analysis of variance (ANOVA).

### Availability of data.

The transcriptome (RNA-seq) sequence data for this study have been submitted to the European Nucleotide Archive (ENA). The accession numbers can be found in Table S1.

10.1128/mBio.01347-18.2 TEXT S2  Expanded supplemental figure legends. Download TEXT S2, DOCX file, 0.1 MB. Copyright © 2018 Liu et al. 2018 Liu et al. This content is distributed under the terms of the Creative Commons Attribution 4.0 International license.
