# Supplementary material for: Investigating the Campylobacter jejuni Transcriptional Response to Host Intestinal Extracts Reveals the Involvement of a Widely Conserved Iron Uptake System
Source: mBio. 2018 Aug 7;9(4):e01347-18. doi: 10.1128/mBio.01347-18 (PMC6083913; doi:10.1128/mBio.01347-18)
Supplement: TABLE S1 [file mbo004183991st1.docx]

Table S1. Conditions for RNA sequencing, sequencing depth, accession numbers, and definition of sample grouping for fold change analysis

| **Exposure Time** | **Exposure Condition** | **Replicate** | **Total # of clusters read** | **% mapped to *C. jejuni* 81-176 genome** | **RNA Sequencing Accession ID** | | | **Comparative Condition Groups** | |
| --- | --- | --- | --- | --- | --- | --- | --- | --- | --- |
|  |  |  |  |  | **Sample Name** | **Sequencing ID** | **Accession number** | **Extract vs. Media** | **Human vs. Chicken** |
| **20 Min** | MH only | 1 | 5891035 | 84.8 | MH_30_A | 16976_1#1 | ERR2004664 | **"Media"** | *Not Used* |
|  | MH only | 2 | 6519638 | 81.2 | MH_30_B | 16976_1#15 | ERR2004678 |  |  |
|  | MH + 30% CP | 1 | 6678478 | 91.4 | CP_30_A | 16976_1#2 | ERR2004665 | **"Extract"** | **"Chicken"** |
|  | MH + 30% CP | 2 | 6974565 | 83.6 | CP_30_B | 16976_1#16 | ERR2004679 |  |  |
|  | MH + 30% HP1 | 1 | 6370997 | 91.2 | HP1_30A | 16976_1#3 | ERR2004666 |  | **"Human"** |
|  | MH + 30% HP1 | 2 | 6009129 | 88.0 | HP1_30B | 16976_1#17 | ERR2004680 |  |  |
|  | MH + 30% HP2 | 1 | 6196054 | 91.3 | HP2_30_A | 16976_1#4 | ERR2004667 |  |  |
|  | MH + 30% HP2 | 2 | 6138116 | 87.3 | HP2_30_B | 16976_1#18 | ERR2004681 |  |  |
|  | MH + 30% HP3 | 1 | 7218437 | 91.0 | HP3_30_A | 16976_1#5 | ERR2004668 |  |  |
|  | MH + 30% HP3 | 2 | 6454389 | 85.3 | HP3_30_B | 16976_1#19 | ERR2004682 |  |  |
| **5 H** | MH only | 1 | 6816668 | 82.6 | MH_5_A | 16976_1#6 | ERR2004669 | **"Media"** | *Not Used* |
|  | MH only | 2 | 6955298 | 78.3 | MH_5_B | 16976_1#20 | ERR2004683 |  |  |
|  | MH + 30% CP | 1 | 7198393 | 87.6 | CP_5_A | 16976_1#7 | ERR2004670 | **"Extract"** | **"Chicken"** |
|  | MH + 30% CP | 2 | 6684505 | 86.5 | CP_5_B | 16976_1#21 | ERR2004684 |  |  |
|  | MH + 30% HP1 | 1 | 7219901 | 89.7 | HP1_5A | 16976_1#8 | ERR2004671 |  | **"Human"** |
|  | MH + 30% HP1 | 2 | 6559664 | 87.9 | HP1_5B | 16976_1#22 | ERR2004685 |  |  |
|  | MH + 30% HP2 | 1 | 6846296 | 88.7 | HP2_5_A | 16976_1#9 | ERR2004672 |  |  |
|  | MH + 30% HP2 | 2 | 6895487 | 87.8 | HP2_5_B | 16976_1#23 | ERR2004686 |  |  |
|  | MH + 30% HP3 | 1 | 7541489 | 88.8 | HP3_5_A | 16976_1#10 | ERR2004673 |  |  |
|  | MH + 30% HP3 | 2 | 7942487 | 87.4 | HP3_5_B | 16976_1#24 | ERR2004687 |  |  |
