# Supplementary figures and images for: Investigating the Campylobacter jejuni Transcriptional Response to Host Intestinal Extracts Reveals the Involvement of a Widely Conserved Iron Uptake System
Source: mBio. 2018 Aug 7;9(4):e01347-18. doi: 10.1128/mBio.01347-18 (PMC6083913; doi:10.1128/mBio.01347-18)

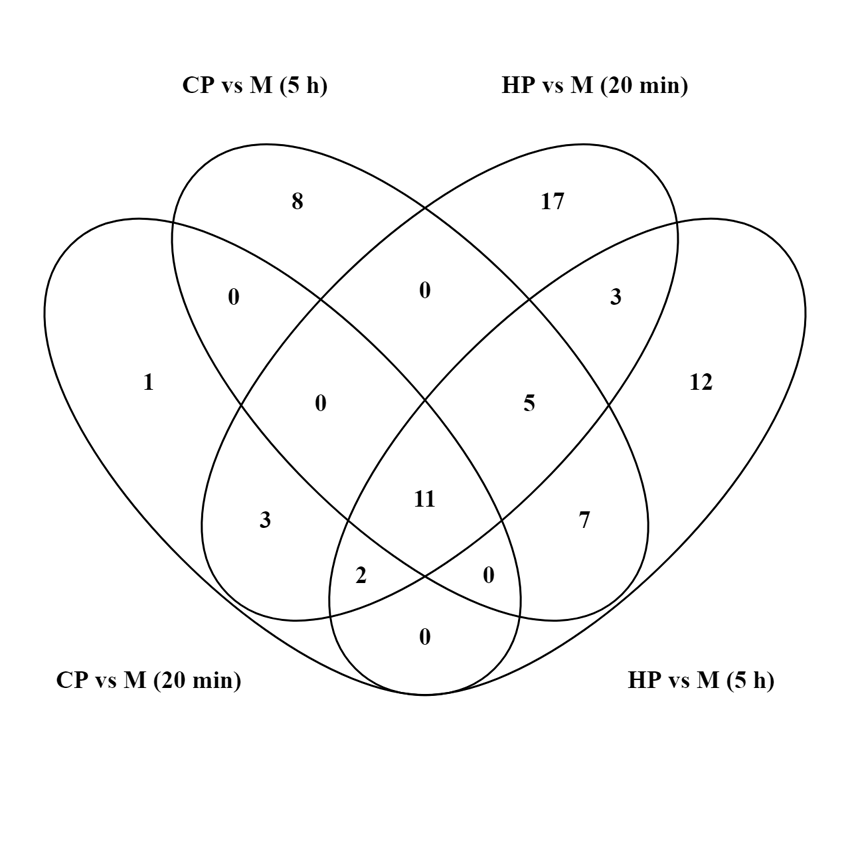

Supplement: FIG S1 [file mbo004183991sf1.tif]

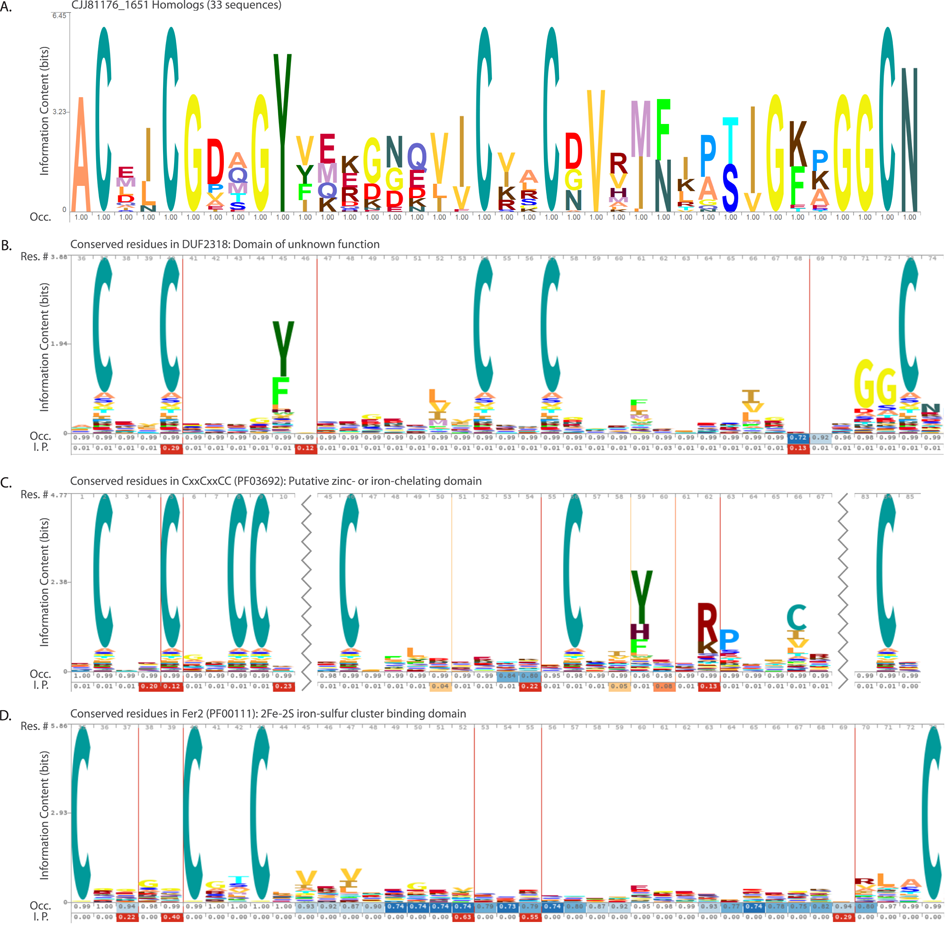

Supplement: FIG S3 [file mbo004183991sf3.tif]
