# Supplementary material for: Investigating the Campylobacter jejuni Transcriptional Response to Host Intestinal Extracts Reveals the Involvement of a Widely Conserved Iron Uptake System
Source: mBio. 2018 Aug 7;9(4):e01347-18. doi: 10.1128/mBio.01347-18 (PMC6083913; doi:10.1128/mBio.01347-18)
Supplement: TABLE S2 [file mbo004183991st2.docx]

Table S2. Changes in gene expression of *C. jejuni* cultured in the presence of chicken extracts vs. media alone and human extracts vs. media alone

|  |  |  | **Chicken vs. Media** | | | |  | **Human vs. Media** | | | |
| --- | --- | --- | --- | --- | --- | --- | --- | --- | --- | --- | --- |
| **Locus Tag** | **Gene Name** | **Gene Product Function** | **20 Minute** | | **5 Hour** | |  | **20 Minute** | | **5 Hour** | |
|  |  |  | **Fold Change** | **P-value** | **Fold Change** | **P-value** |  | **Fold Change** | **P-value** | **Fold Change** | **P-value** |
| **Genes with increased expression** | | | | | | | | | | | |
| **Metabolism, nutrient uptake, and energy production** | | |  |  |  |  |  |  |  |  |  |
| CJJ81176_0122 | *aspA* | aspartate ammonia-lyase | **3.99** | 6.29E-05 | **2.96** | 4.60E-03 |  | **3.47** | 2.02E-06 | **2.70** | 3.91E-04 |
| CJJ81176_0123 | *dcuA* | anaerobic C4-dicarboxylate membrane transporter DcuA | **3.54** | 4.52E-06 | **2.83** | 3.54E-04 |  | **3.01** | 1.60E-07 | **2.58** | 1.48E-05 |
| CJJ81176_0438 | *CJJ81176_0438* | putative oxidoreductase subunit | **4.33** | 3.51E-45 | **10.33** | 1.59E-116 |  | **4.74** | 4.68E-77 | **13.09** | 6.22E-212 |
| CJJ81176_0439 | *CJJ81176_0439* | oxidoreductase, putative | **4.25** | 4.32E-17 | **10.94** | 4.70E-48 |  | **4.85** | 3.26E-31 | **13.99** | 1.70E-88 |
| CJJ81176_0467 | *CJJ81176_0467* | hypothetical protein |  |  |  |  |  | **2.15** | 2.31E-02 |  |  |
| CJJ81176_0697 | *dcuB* | anaerobic C4-dicarboxylate membrane transporter DcuB | **2.32** | 2.04E-05 | **3.24** | 3.87E-11 |  | **2.06** | 1.85E-06 | **3.15** | 2.96E-16 |
| CJJ81176_0884 | *CJJ81176_0884* | cytochrome c family protein, degenerate |  |  | **2.85** | 1.95E-09 |  | **2.14** | 1.25E-07 | **3.09** | 7.23E-17 |
| CJJ81176_0885 | *CJJ81176_0885* | cytochrome C | **2.26** | 1.16E-04 | **3.98** | 5.08E-14 |  | **2.52** | 1.90E-09 | **4.47** | 3.01E-25 |
| CJJ81176_1242 | *htrA* | protease DO |  |  |  |  |  | **2.27** | 6.27E-03 |  |  |
| CJJ81176_1389 | *CJJ81176_1389* | DNA-binding protein |  |  |  |  |  | **2.27** | 3.37E-05 |  |  |
| CJJ81176_1390 | *CJJ81176_1390* | reactive intermediate/imine deaminase |  |  |  |  |  | **5.70** | 3.07E-03 |  |  |
| CJJ81176_1391 | *CJJ81176_1391* | C4-dicarboxylate ABC transporter |  |  |  |  |  | **4.77** | 5.19E-04 |  |  |
| CJJ81176_1392 | *metC* | cystathionine beta-lyase |  |  |  |  |  | **4.33** | 1.34E-04 |  |  |
| CJJ81176_1393 | *purB-2* | adenylosuccinate lyase |  |  |  |  |  | **3.80** | 8.49E-04 |  |  |
| CJJ81176_1394 | *CJJ81176_1394* | MmgE/PrpD family protein |  |  |  |  |  | **3.21** | 5.45E-03 |  |  |
| CJJ81176_1501 | *fdhC* | formate dehydrogenase. cytochrome b subunit |  |  | **2.29** | 4.60E-03 |  |  |  |  |  |
| CJJ81176_1502 | *fdhB* | formate dehydrogenase. iron-sulfur subunit |  |  | **2.23** | 3.44E-03 |  |  |  |  |  |
| CJJ81176_1570 | *CJJ81176_1570* | anaerobic dimethyl sulfoxide reductase chain A |  |  |  |  |  |  |  | **3.11** | 5.27E-03 |
| **Iron uptake** | | |  |  |  |  |  |  |  |  |  |
| CJJ81176_1649 | *CJJ81176_1649* | iron permease, FTR1 family | **3.00** | 2.41E-02 |  |  |  | **2.91** | 1.07E-03 | **3.76** | 2.01E-05 |
| CJJ81176_1650 | *p19* | periplasmic iron binding protein | **2.86** | 4.37E-02 |  |  |  | **3.11** | 5.19E-04 | **4.61** | 6.73E-07 |
| CJJ81176_1651 | *CJJ81176_1651* | membrane protein, putative |  |  |  |  |  |  |  | **3.71** | 1.06E-04 |
| CJJ81176_1652 | *CJJ81176_1652* | ABC transporter, permease protein |  |  |  |  |  |  |  | **4.16** | 3.98E-05 |
| CJJ81176_1653 | *CJJ81176_1653* | ABC transporter, permease protein |  |  |  |  |  | **2.68** | 8.98E-03 | **4.77** | 1.45E-06 |
| CJJ81176_1654 | *CJJ81176_1654* | ABC transporter, ATP-binding protein |  |  |  |  |  |  |  | **5.38** | 2.74E-07 |
| CJJ81176_1655 | *CJJ81176_1655* | thiredoxin, homolog |  |  |  |  |  |  |  | **5.50** | 2.97E-06 |
| **Hypothetical Proteins** | | |  |  |  |  |  |  |  |  |  |
| CJJ81176_0204 | *CJJ81176_0204* | hypothetical protein | **2.79** | 8.75E-09 | **3.58** | 2.79E-14 |  | **3.10** | 7.54E-17 | **5.06** | 8.03E-36 |
| CJJ81176_0440 | *CJJ81176_0440* | conserved hypothetical protein | **2.38** | 1.07E-05 | **2.01** | 8.44E-04 |  | **2.55** | 1.68E-10 | **2.26** | 3.90E-08 |
| CJJ81176_1005 | *CJJ81176_1005* | membrane protein, putative | **2.45** | 8.24E-03 |  |  |  | **2.10** | 6.07E-03 |  |  |
| **Unknown** | | |  |  |  |  |  |  |  |  |  |
| CP000538_intergenic_386739_386926 | |  | **2.55** | 1.75E-21 | **2.59** | 3.13E-22 |  | **2.92** | 3.74E-41 | **3.02** | 7.98E-45 |
| CP000538_intergenic_1565120_1565213 | |  |  |  |  |  |  |  |  | **4.81** | 6.31E-06 |
| CP000538_intergenic_1580627_1580720 | |  |  |  |  |  |  | **2.20** | 4.99E-02 |  |  |
|  |  |  |  |  |  |  |  |  |  |  |  |
| **Genes with reduced expression** | | | | | | | | | | | |
| **Adhesin** |  |  |  |  |  |  |  |  |  |  |  |
| CJJ81176_0315 | *peb3* | major antigenic peptide PEB3 | **-3.36** | 3.10E-03 | **-7.54** | 2.19E-09 |  | **-2.88** | 4.27E-04 | **-8.44** | 3.90E-16 |
| **Metabolism, nutrient uptake, and energy production** | | |  |  |  |  |  |  |  |  |  |
| CJJ81176_0033 | *gltB* | glutamate synthase, large subunit |  |  |  |  |  |  |  | **-2.35** | 2.79E-04 |
| CJJ81176_0035 | *gltD* | glutamate synthase, small subunit |  |  | **-2.01** | 2.33E-18 |  |  |  | **-2.19** | 4.40E-36 |
| CJJ81176_0120 | *CJJ81176_0120* | aspartate racemase. putative | **-2.25** | 2.37E-04 |  |  |  |  |  |  |  |
| CJJ81176_0580 | *CJJ81176_0580* | C4-dicarboxylate ABC transporter |  |  | **-3.59** | 3.87E-11 |  | **-2.02** | 3.84E-05 | **-3.62** | 2.25E-17 |
| CJJ81176_0581 | *CJJ81176_0581* | amidohydrolase |  |  | **-2.18** | 2.68E-04 |  |  |  | **-2.05** | 6.72E-06 |
| CJJ81176_0642 | *CJJ81176_0642* | phosphate ABC transporter. periplasmic phosphate-binding protein. |  |  |  |  |  |  |  | **-2.21** | 8.12E-03 |
| CJJ81176_0643 | *CJJ81176_0643* | phosphate ABC transporter. permease protein PstC. |  |  |  |  |  |  |  | **-2.23** | 1.15E-02 |
| CJJ81176_0644 | *CJJ81176_0644* | phosphate ABC transporter. permease protein PstA. |  |  |  |  |  | **-2.04** | 1.01E-02 |  |  |
| CJJ81176_0685 | *CJJ81176_0685* | Di-/tripeptide transporter |  |  | **-2.52** | 7.33E-15 |  |  |  | **-3.19** | 8.88E-37 |
| CJJ81176_0722 | *glnA* | glutamine synthetase. type I |  |  |  |  |  |  |  | **-2.26** | 5.33E-03 |
| CJJ81176_0752 | *CJJ81176_0752* | putative type I phosphodiesterase/nucleotide pyrophosphatase |  |  |  |  |  | **-2.10** | 1.10E-02 |  |  |
| CJJ81176_0912 | *CJJ81176_0912* | amino acid carrier protein |  |  | **-2.39** | 6.86E-04 |  |  |  | **-2.79** | 1.40E-08 |
| CJJ81176_0941 | *CJJ81176_0941* | sodium:alanine symporter | **-2.67** | 8.24E-04 |  |  |  | **-2.45** | 4.22E-05 |  |  |
| CJJ81176_0942 | *CJJ81176_0942* | sodium:alanine symporter | **-2.89** | 4.93E-04 |  |  |  | **-2.51** | 6.77E-05 |  |  |
| CJJ81176_1356 | *CJJ81176_1356* | RelE/ParE family plasmid stabilization system protein |  |  |  |  |  | **-2.73** | 2.06E-02 |  |  |
| CJJ81176_1381 | *selB* | selenocysteine-specific elongation factor |  |  | **-2.13** | 2.30E-02 |  |  |  |  |  |
| **Iron uptake** | |  |  |  |  |  |  |  |  |  |  |
| CJJ81176_0210 | *cfbpB* | iron ABC transporter. permease protein |  |  | **-3.55** | 1.64E-02 |  |  |  |  |  |
| CJJ81176_0211 | *cfbpA* | iron ABC transporter, periplasmic iron-binding protein |  |  | **-4.11** | 1.69E-02 |  |  |  |  |  |
| **Chemotaxis** | | |  |  |  |  |  |  |  |  |  |
| CJJ81176_0109 | *CJJ81176_0109* | methyl-accepting chemotaxis protein |  |  |  |  |  |  |  | **-2.80** | 1.77E-03 |
| CJJ81176_0289 | *CJJ81176_0289* | methyl-accepting chemotaxis protein |  |  |  |  |  | **-2.01** | 7.22E-08 |  |  |
| **Oxidative Response** | | |  |  |  |  |  |  |  |  |  |
| CJJ81176_0266 | *herA* | hemerythrin |  |  |  |  |  | **-2.30** | 4.42E-02 | **-2.47** | 3.12E-02 |
| CJJ81176_1492 | *fdhT* | membrane protein, putative |  |  | **-12.83** | 2.78E-09 |  |  |  |  |  |
| CJJ81176_1493 | *fdhU* | conserved hypothetical protein |  |  | **-4.40** | 5.46E-04 |  |  |  |  |  |
| CJJ81176_1656 | *CJJ81176_1656* | thioredoxin family protein |  |  |  |  |  | **-2.13** | 3.31E-02 |  |  |
| **Hypothetical Proteins** | | |  |  |  |  |  |  |  |  |  |
| CJJ81176_0034 | *CJJ81176_0034* | hypothetical protein |  |  |  |  |  |  |  | **-2.03** | 4.24E-12 |
| CJJ81176_0522 | *CJJ81176_0522* | hypothetical protein |  |  |  |  |  | **-2.86** | 3.03E-02 |  |  |
| CJJ81176_1006 | *CJJ81176_1006* | hypothetical protein |  |  | **-2.63** | 4.74E-02 |  |  |  | **-2.69** | 1.90E-03 |
| CJJ81176_1184 | *CJJ81176_1184* | hypothetical protein |  |  | **-2.83** | 2.27E-02 |  | **-2.10** | 2.68E-02 | **-2.72** | 1.44E-03 |
| CJJ81176_1185 | *CJJ81176_1185* | hypothetical protein |  |  | **-3.22** | 3.18E-02 |  |  |  | **-3.00** | 3.59E-03 |
| CJJ81176_1657 | *CJJ81176_1657* | hypothetical protein | **-2.61** | 3.51E-24 | **-2.37** | 1.30E-19 |  | **-2.71** | 2.63E-40 | **-2.88** | 1.35E-45 |
| CJJ81176_pVir0035 | *CJJ81176_pVir0035* | hypothetical protein |  |  | **-4.64** | 3.79E-02 |  |  |  | **-3.94** | 4.67E-03 |
| **Unknown** | | |  |  |  |  |  |  |  |  |  |
| CP000538_intergenic_8059_8091 | |  |  |  |  |  |  | **-2.35** | 2.17E-02 |  |  |
| CP000538_intergenic_94609_94680 | |  |  |  | **-4.16** | 4.51E-02 |  | **-2.96** | 4.32E-02 | **-3.16** | 1.32E-02 |
| CP000538_intergenic_530014_530163 | |  |  |  |  |  |  | **-2.13** | 1.02E-02 | **-2.37** | 2.93E-03 |
| CP000538_intergenic_610942_611068 | |  |  |  |  |  |  | **-3.39** | 3.76E-03 |  |  |
| CP000538_intergenic_859156_859190 | |  |  |  | **-2.84** | 4.93E-02 |  |  |  |  |  |
| CP000538_intergenic_1272541_1272799 | |  |  |  | **-2.89** | 2.01E-04 |  | **-2.22** | 2.05E-04 | **-3.05** | 3.89E-08 |
