# Supplementary material for: Investigating the Campylobacter jejuni Transcriptional Response to Host Intestinal Extracts Reveals the Involvement of a Widely Conserved Iron Uptake System
Source: mBio. 2018 Aug 7;9(4):e01347-18. doi: 10.1128/mBio.01347-18 (PMC6083913; doi:10.1128/mBio.01347-18)
Supplement: TABLE S3 [file mbo004183991st3.docx]

Table S3. Homologs of the *C. jejuni* 1649 - 1656 system

| **Organism (in alphabetical order)** | **Homologous genes** | | | **Amino acid sequence compared to *C. jejuni* 81-176 (% identity)** | | | | | | |
| --- | --- | --- | --- | --- | --- | --- | --- | --- | --- | --- |
|  |  |  |  | **1649** | **1650** | **1651** | **1652** | **1653** | **1654** | **1655** |
| *Actinobacillus succinogenes* 130Z | ASUC_RS08610 | - | ASUC_RS08645 | 31% | 55% | 26% | 38% | 32% | 56% | 24% |
| *Aggregatibacter actinomycetemcomitans* NUM4039 | AANUM_1503 | - | AANUM_1496 | 31% | 54% | 25% | 36% | 33% | 61% | 21% |
| *Bifidobacterium kashiwanohense* PV20-2 | AH68_00590 | - | AH68_00620 | 17% | 31% | 16% | 27% | 22% | 42% | N/A |
| *Brenneria goodwinii* | BN1221_04853c | - | BN1221_04847c | 29% | 47% | 25% | 35% | 31% | 54% | 25% |
| *Campylobacter coli* RM4661 | YSS_RS00485 | - | YSS_RS00450 | 94% | 100% | 86% | 91% | 87% | 90% | 77% |
| *Campylobacter fetus* 82-40 | CFF8240_RS02595 | - | CFF8240_RS02625 | 37% | 64% | 34% | 51% | 40% | 61% | 22% |
| *Campylobacter lari* RM2100 | CLA_RS05835 | - | CLA_RS05800 | 63% | 75% | 46% | 58% | 60% | 74% | 42% |
| *Citrobacter koseri* ATCC BAA-895 | CKO_RS08730 | - | CKO_RS08695 | 30% | 51% | 26% | 36% | 30% | 57% | 23% |
| *Escherichia coli* VR50 plasmid pVR50B | ECVR50_B070 | - | ECVR50_B063 | 29% | 51% | 26% | 36% | 30% | 57% | 23% |
| *Filifactor alocis* ATCC 35896 | HMPREF0389_RS08245 | - | HMPREF0389_RS08215 | 17% | 31% | 16% | 31% | 23% | 52% | N/A |
| *Flexistipes sinusarabici* DSM 4947 | FLEXSI_RS09550 | - | FLEXSI_RS09525 | 19% | 49% | 17% | 33% | 28% | 52% | N/A |
| *Frischella perrara* PEB0191 | FPB0191_RS04035 | - | FPB0191_RS04065 | 19% | 38% | 16% | 32% | 27% | 56% | N/A |
| *Haemophilus parainfluenzae* T3T1 | PARA_17390 | - | PARA_17460 | 31% | 53% | 26% | 38% | 34% | 58% | 28% |
| *Jonquetella anthropi* DSM 22815 | JONANDRAFT_RS04580 | - | JONANDRAFT_RS04610 | 18% | 32% | 15% | 28% | 24% | 51% | N/A |
| *Klebsiella pneumoniae* B2 plasmid pB2-A/C | AOG75827.1 | - | AOG75834.1 | 29% | 51% | 26% | 36% | 30% | 57% | N/A |
| *Leminorella grimontii* ATCC 33999 | G544_RS0102515 | - | G544_RS0102550 | 30% | 51% | 26% | 35% | 33% | 53% | 24% |
| *Mobiluncus curtisii* ATCC 43063 | HMPREF0573_RS07020 | - | HMPREF0573_RS07055 | 17% | 26% | 15% | 31% | 21% | 45% | N/A |
| *Paracoccus halophilus* | IT41_RS00290 | - | IT41_RS00260 | 28% | 50% | 26% | 37% | 32% | 53% | 25% |
| *Parascardovia denticolens* DSM 10105 | HMPREF0620_1658 | - | HMPREF0620_1664 | 18% | 32% | 16% | 29% | 22% | 40% | N/A |
| *Parvimonas micra* KCOM 1535 | NW74_RS03260 | - | NW74_RS03290 | 17% | 27% | 16% | 30% | 27% | 53% | N/A |
| *Pasteurella multocida* Pm70 | PM_RS02335 | - | PM_RS02300 | 31% | 56% | 25% | 37% | 33% | 57% | 24% |
| *Pectobacterium carotovorum* PC1 | PC1_RS06925 | - | PC1_RS06955 | 29% | 53% | 25% | 35% | 31% | 54% | 23% |
| *Pelosinus* sp. UFO1 | UFO1_RS01255 | - | UFO1_RS01285 | 18% | 39% | 16% | 30% | 27% | 54% | N/A |
| *Rhodospirillum rubrum* ATCC 11170 | Rru_A2809 | - | Rru_A2803 | 28% | 55% | 26% | 36% | 30% | 54% | 24% |
| *Salmonella enterica* serovar Typhimurium T000240 | STMDT12_C39040 | - | STMDT12_C38970 | 29% | 51% | 26% | 36% | 30% | 57% | 23% |
| *Selenomonas sputigena* ATCC 35185 | SELSP_RS01220 | - | SELSP_RS01250 | 16% | 42% | 15% | 32% | 25% | 49% | N/A |
| *Shigella dysenteriae* 80-547 plasmid p80-547 | AMQ11474.1 | - | AMQ11475.1 | 29% | 51% | 26% | 36% | 30% | 57% | 23% |
| *Spirochaeta thermophila* DSM 6578 | SPITH_RS11755 | - | SPITH_RS02265 | 16% | 51% | 18% | 32% | 26% | 58% | N/A |
| *Sulfurospirillum multivorans* DSM 12446 | SMUL_2708 | - | SMUL_2716 | 39% | 56% | 36% | 50% | 45% | 65% | 29% |
| *Treponema* sp. OMZ 838 | JO41_09660 | - | JO41_09685 | 16% | 32% | 17% | 32% | 21% | 53% | N/A |
| *Wolinella succinogenes* DSM 1740 | WS1566 | - | WS1557 | 38% | 58% | 36% | 51% | 44% | 65% | 33% |
| *Yersinia pestis* CO92 | YPO1941 | - | YPO1948 | 30% | 55% | 26% | 35% | 29% | 54% | 25% |
| **Minimum** | | | | **16%** | **26%** | **15%** | **27%** | **21%** | **40%** | **21%** |
| **Maximum** | | | | **94%** | **100%** | **86%** | **91%** | **87%** | **90%** | **77%** |
