# Supplementary material for: Investigating the Campylobacter jejuni Transcriptional Response to Host Intestinal Extracts Reveals the Involvement of a Widely Conserved Iron Uptake System
Source: mBio. 2018 Aug 7;9(4):e01347-18. doi: 10.1128/mBio.01347-18 (PMC6083913; doi:10.1128/mBio.01347-18)
Supplement: TEXT S1 [file mbo004183991s1.docx]

Text S1. Supplemental methods

**Construction of the *cjj81176_1651*-*1656* mutant and complement.** The *1651*-*1656* deletion *(∆1651-1656*) was prepared using a modified Gibson assembly protocol with the NEBuilder HiFi DNA Assembly kit (NEB). A total of 676 bases upstream of *1651* and 535 bases downstream of *1656* was PCR amplified using iProof DNA polymerase (BioRad) with *C. jejuni* 81-176 genomic DNA and primers ML1651u5’ and ML1651gu3’, and ML1655gd5’ and ML1655d3’ (Table S3). Primers ML1651gu3’ and ML1655gd5’ were designed to have complementary overhangs to allow attachment of the kanamycin resistance (Km^R^) cassette. The Km^R^ cassette was amplified from the plasmid pRRK (obtained from J. Ketley) with iProof polymerase and primers MLkanRgu5’ and MLkanRgd3’ (Table S3). The three PCR fragments were purified using the DNA Clean & Concentrator Kit (Zymo Research) and annealed as per manufacturer’s directions (NEBuilder). The resulting annealed fragment was PCR amplified using primers ML1651u5’ and ML1655d3’ and purified. The PCR product was transformed into *C. jejuni* by natural transformation and selected for on MH-Km. The genomic deletion was confirmed by PCR using primers ML1651u5’ and ML1655d3’ (Table S3).

To complement ∆*1651-1656,* the *1651*-*1656* gene region was PCR amplified using iProof High Fidelity DNA Polymerase (BioRad) from *C. jejuni* 81-176 genomic DNA using primers ML165116565’ and ML165116563’ (Table S3). The PCR product and the pRRC plasmid, which is used to insert genes of interest between the 16S rRNA and tRNA region in the *C. jejuni* chromosome (1), were digested using *XbaI* (NEB), ligated, and transformed into *E. coli* DH5α. Transformants were selected on LB-Cm. The recombinant plasmid was verified by PCR and sequencing, and then transformed into *C. jejuni* *Δ1651-1656* by natural transformation and plating on MH-Cm. The complemented strain (designated *1651-1656^c^*) was verified by PCR and sequencing.

**Sample collection and extract preparation.** Chicken gut pouches were obtained from 36 day old Ross 308 Broilers at Sunrise Poultry Processors Ltd in Surrey, British Columbia. Samples were taken directly off the processing line where the unbroken gut sacs (crop to anus) were removed intact from the recently slaughtered chickens. The gut sacs were severed between the gizzard and the duodenum, and the portion containing the ceca was packed in individual bags and transported on ice. Cecal material was extracted by making an incision at the distal tip of each cecal pouch and transferring the cecal material directly into pre-weighed 50 mL tubes. The material from both cecal pouches per chicken was pooled and weighed, and extract was prepared within 20 h of receiving the samples.

Human fecal samples were collected from healthy volunteers, defined as people who did not have chronic gastrointestinal disorders (such as inflammatory bowel disease, Celiac disease, Crohn’s disease, or intestinal cancer), who were not taking drugs that might impact the intestinal microbiome (such as antibiotics or immune modifying drugs), and who had not had an episode of diarrhea within 5 days prior to donation. These exclusionary criteria were implemented to reduce variables that would disrupt the intestinal microbiome, or introduce drugs or antibodies that may kill *C. jejuni* added to the extract. Samples were collected from both male and female volunteers aged between 22 and 51 (Fig. 1A). Fecal samples were collected directly into the specimen collection system (Fisher Scientific) and refrigerated within 20 min of collection. Each sample was given a study identifier (H = human, # = number identifier) and transferred into pre-weighed 50 mL tubes prior to extract preparation. Extract was prepared within 8 h of receiving the fecal samples.

Chicken cecal and human fecal samples were weighed and diluted using sterile water at a ratio of 1:1 to 1:1.5 (w/v) depending on dryness and solidity of the material in order to allow enough liquid for filter sterilization and to ensure consistency of final homogenate. 8-15 glass beads (4 mm diameter) were added to the mixture, vortexed at maximum speed to break up large clumps, and then mixed by inversion at 4°C for 30 min. The tubes were centrifuged at 10,000 x g for 30 min and the supernatant was transferred to a new tube. Centrifuging was repeated 3-6 x as necessary to remove solid materials and mucus. The resulting supernatant was filter sterilized and stored in 1 mL aliquots at -20°C. Samples were pooled to create a CP (Chicken Pool) and HP1/2/3 (Human Pools 1, 2 and 3) as noted in Fig. 1A, and the pH of the pooled extracts was measured using a pH meter (SB20, VWR). Sterility of pooled extracts was confirmed by no observable bacterial or fungal growth after inoculation of a drop of extract onto the surface of MH and LB plates and incubating them microaerobically at 38°C and aerobically at 37°C, respectively (data not shown).

**Biofilm quantification**. 250 µL crystal violet (1% w/v in ethanol) was added to the biofilm culture tubes, and the mixture was incubated for 10-15 min at room temperature. The tubes were washed 3 times with H_2_O, dried overnight, and destained by adding 1.5 mL of destaining solution (30% methanol and 10% acetic acid in H_2_O) for 24 h. The total amount of biofilm was quantified by measuring the absorbance at 570 nm using a Varioscan Flash Spectrophotometer (Thermo Scientific).

**RNA extraction, sequencing, and data analysis.** RNA purity was verified using the Agilent Bioanalyzer, and samples were subjected to ribosomal RNA depletion (Ribo-Zero™ Magnetic kit, Epicentre). The samples were sequenced using the TruSeq non-stranded protocol. The samples were run on the Illumina HiSeq 2500 platform with >600 fold coverage. The resulting FASTQ reads were mapped onto the *C. jejuni* 81-176 genome (Genbank accession number NC_008787.1) using SMALT v0.75 (http://www.sanger.ac.uk/science/tools/smalt-0). The read counts per CDS feature was used for differential expression analysis using the DESeq2 package in R. The counts were adjusted using the rlog transformation to compare genes with low read count. Differential gene expression was determined for 20 min and 5 h exposures of *C. jejuni* exposed to media + human extract (HP1, HP2, and HP3) in comparison to media + chicken extract (CP) (i.e. human vs. chicken), and media + extract (HP1, HP2, HP3, CP) in comparison to media alone (i.e. extract vs. media) as grouped according to Table S1. Note, RNA sequencing and analysis was completed prior to Genbank update of the *C. jejuni* 81-176 genome annotation, therefore the previous locus identifiers are used throughout this study.

SUPPLEMENTAL REFERENCES

1. Karlyshev AV, Wren BW. 2005. Development and application of an insertional system for gene delivery and expression in Campylobacter jejuni. Appl Environ Microbiol 71:4004-13.
