# Supplementary material for: Investigating the Campylobacter jejuni Transcriptional Response to Host Intestinal Extracts Reveals the Involvement of a Widely Conserved Iron Uptake System
Source: mBio. 2018 Aug 7;9(4):e01347-18. doi: 10.1128/mBio.01347-18 (PMC6083913; doi:10.1128/mBio.01347-18)
Supplement: TABLE S4 [file mbo004183991st4.docx]

Table S4. Primers used in this study

| **Primer ID** | **Description** | **Primer Sequence** | **Restriction Site** |
| --- | --- | --- | --- |
| **For Deletion Strain** | | | |
| ML1651u5' | Upstream of *CJJ81176_1651* | GAACCGATTTTATGGCTTGG | N/A |
| ML1651gu3' | Upstream of *CJJ81176_1651* with overlap to attach KmR | GTCGACCTCGACTAGAACACTCGGAAAATCCGAGTGTAAAATCATTTTGGCGTGCCTGTG | N/A |
| MLkanRgu5' | KmR with upstream overlap | GATTTTCCGAGTGTTCTAGTCGAGGTCGACGGTATCGATAAGCTTGATATCGAATTCCTG | N/A |
| MLkanRgd3' | KmR with downstream overlap | ATGGCACTTGAAAGGGAACTAGTGGATCCCGGCCTCAGGCACGCAAGCTTTTTAGACATC | N/A |
| ML1655gd5' | Downstream of *CJJ81176_1655* with overlap to attach KmR | GGGATCCACTAGTTCCCTTTCAAGTGCCATTGGGGAAATATATGGAGTGCCTGTGCTTAG | N/A |
| ML1655d3' | Downstream of *CJJ81176_1655* | CTCACTCTTACGCAAGCTAAG | N/A |
| **For Complement** | | | |
| ML165116565' | Upstream of gene *CJJ81176_1651* | CATTCTAGAGCCATGTTGATGAAGAAACAG | *XbaI* |
| ML165116563' | Downstream of gene *CJJ81176_1656* | CATTCTAGAGATGGAAGCTATGAGCTTTATGG | *XbaI* |
