# Supplementary material for: Investigating the Campylobacter jejuni Transcriptional Response to Host Intestinal Extracts Reveals the Involvement of a Widely Conserved Iron Uptake System
Source: mBio. 2018 Aug 7;9(4):e01347-18. doi: 10.1128/mBio.01347-18 (PMC6083913; doi:10.1128/mBio.01347-18)
Supplement: TEXT S2 [file mbo004183991s2.docx]

Supplemental Figure Legends

FIG S1. Venn diagram of the number of genes differently expressed during growth in the presence of chicken extracts (CP) vs. media alone (M) and human extracts (HP) vs. media alone (M) after 20 min and 5 h. The list of genes is provided in Table S2. Generated using Venny (2.1) (1).

FIG S2. Growth of *C. jejuni* strains in iron depleted media and with extract supplementation over 48 h. *C. jejuni* growth in control media (MH; black), iron limited media (MH + 15 µM DFO; gray), and iron limited media supplemented with extracts (10% CP/HP1/HP2/HP3; CP = pink, HP1 = light blue, HP2 = medium blue, HP3 = dark blue) or iron(III) citrate (10 µM; black dashed/striped). Growth as measured by OD 600 nm (A, B, C), representing total accumulated growth, and by dilution plating (D, E, F), representing cell viability, at set-up (0 h; A and D), after 24 h incubation (B and E), and after 48 h incubation (C and F).

FIG S3. The CxxC-x(13)-CxxC-x(14,15)-C motif of the DUF2318 domain in 1651. (A) The conserved CxxC-x(13)-CxxC-x(14)-C region of the 33 homologs of 1651 as listed in Table S2 analyzed with SkyLign. (B) The Hidden Markov Model (HMM) of conserved cysteine residues in the DUF2318 family as output from EMBL-EBI (2). (C and D) HMMs of conserved cysteine residues from the Fer2 (PF00111) and Fer4 (PF00037) domains, which are involved in iron-sulfur cluster binding. In the conserved residue plots the number of different letters in one position indicates the number of different amino acids, the height of the letters indicate the degree of residue conservation. Res. # indicates residue number. Occ. represents % occupancy, with blue boxes indicating the proportion of sequences missing that residue. I.P. represents insert probability, with red boxes indicate proportion of sequences with insertions occurring at the orange lines.

SUPPLEMENTAL REFERENCES

1. Oliveros JC. 2007-2015. Venny. An interactive tool for comparing lists with Venn's diagrams. <http://bioinfogp.cnb.csic.es/tools/venny/index.html>. Accessed

2. Schuster-Bockler B, Schultz J, Rahmann S. 2004. HMM Logos for visualization of protein families. BMC Bioinformatics 5:7.
